# Multi‐omic landscaping of human midbrains identifies disease‐relevant molecular targets and pathways in advanced‐stage Parkinson's disease

**DOI:** 10.1002/ctm2.692

**Published:** 2022-01-28

**Authors:** Lucas Caldi Gomes, Ana Galhoz, Gaurav Jain, Anna‐Elisa Roser, Fabian Maass, Eleonora Carboni, Elisabeth Barski, Christof Lenz, Katja Lohmann, Christine Klein, Mathias Bähr, André Fischer, Michael P. Menden, Paul Lingor

**Affiliations:** ^1^ Department of Neurology Rechts der Isar Hospital Technical University of Munich München Germany; ^2^ Department of Neurology University Medical Center Göttingen Göttingen Germany; ^3^ Helmholtz Zentrum München GmbH ‐ German Research Center for Environmental Health Institute of Computational Biology Neuherberg Germany; ^4^ Department of Biology Ludwig‐Maximilians University Munich Martinsried Germany; ^5^ Department for Epigenetics and Systems Medicine in Neurodegenerative Diseases German Center for Neurodegenerative Diseases (DZNE) Göttingen Germany; ^6^ Institute of Clinical Chemistry University Medical Center Göttingen Göttingen Germany; ^7^ Bioanalytical Mass Spectrometry Group Max Planck Institute for Biophysical Chemistry Göttingen Germany; ^8^ Institute of Neurogenetics University of Lübeck Lübeck Germany; ^9^ Department of Psychiatry and Psychotherapy University Medical Center Göttingen Göttingen Germany; ^10^ German Centre for Diabetes Research (DZD e.V.) Neuherberg Germany; ^11^ German Center for Neurodegenerative Diseases (DZNE) München Germany

**Keywords:** data integration, miRNAs, multi‐omics, Parkinson disease

## Abstract

**Background:**

Parkinson's disease (PD) is the second most common neurodegenerative disorder whose prevalence is rapidly increasing worldwide. The molecular mechanisms underpinning the pathophysiology of sporadic PD remain incompletely understood. Therefore, causative therapies are still elusive. To obtain a more integrative view of disease‐mediated alterations, we investigated the molecular landscape of PD in human post‐mortem midbrains, a region that is highly affected during the disease process.

**Methods:**

Tissue from 19 PD patients and 12 controls were obtained from the Parkinson's UK Brain Bank and subjected to multi‐omic analyses: small and total RNA sequencing was performed on an Illumina's HiSeq4000, while proteomics experiments were performed in a hybrid triple quadrupole‐time of flight mass spectrometer (TripleTOF5600+) following quantitative sequential window acquisition of all theoretical mass spectra. Differential expression analyses were performed with customized frameworks based on DESeq2 (for RNA sequencing) and with Perseus v.1.5.6.0 (for proteomics). Custom pipelines in R were used for integrative studies.

**Results:**

Our analyses revealed multiple deregulated molecular targets linked to known disease mechanisms in PD as well as to novel processes. We have identified and experimentally validated (quantitative real‐time polymerase chain reaction/western blotting) several PD‐deregulated molecular candidates, including miR‐539‐3p, miR‐376a‐5p, miR‐218‐5p and miR‐369‐3p, the valid miRNA‐mRNA interacting pairs miR‐218‐5p/*RAB6C* and miR‐369‐3p/*GTF2H3*, as well as multiple proteins, such as CHI3L1, HSPA1B, FNIP2 and TH. Vertical integration of multi‐omic analyses allowed validating disease‐mediated alterations across different molecular layers. Next to the identification of individual molecular targets in all explored omics layers, functional annotation of differentially expressed molecules showed an enrichment of pathways related to neuroinflammation, mitochondrial dysfunction and defects in synaptic function.

**Conclusions:**

This comprehensive assessment of PD‐affected and control human midbrains revealed multiple molecular targets and networks that are relevant to the disease mechanism of advanced PD. The integrative analyses of multiple omics layers underscore the importance of neuroinflammation, immune response activation, mitochondrial and synaptic dysfunction as putative therapeutic targets for advanced PD.

## Graphical abstract

Midbrain samples of Parkinson's disease (PD) and control (CTR) patients were subjected to a multi‐omic analysis. The expression of miRNAs, transcripts and proteins was explored, followed by data integration and functional analyses. This comprehensive assessment of PD‐affected and CTR human midbrains revealed multiple molecular targets and networks that are relevant to the disease mechanism of advanced PD.

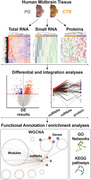

## 1 BACKGROUND

Parkinson's disease (PD) is the fastest‐growing neurodegenerative disorder and affects up to 2% of individuals aged over 60 years.^1^ While the chronic and progressive motor dysfunction is mostly due to degeneration of dopaminergic neurons in the nigrostriatal pathway, PD is now recognized to be a systemic disorder involving multiple other regions of the nervous system.^2^ The sporadic form accounts for most cases of PD, whereas only up to 3% of cases comprise the autosomal forms.^3^ Several environmental and genetic factors are known to increase the disease risk.^4^ Recent studies point to a multifactorial pathogenesis that may differ between patients, suggesting that PD is not one homogeneous disease entity, but rather a syndrome with a unifying clinical phenotype and numerous molecular subgroups. The existence of non‐motor symptoms that appear many years before the onset of motor manifestations suggests that molecular mechanisms may have a long lead‐up period and result in chronic degeneration.^5^ One of the pathological hallmarks of PD is the presence of Lewy‐bodies (LB), intracytoplasmic inclusions that are majorly composed of the protein alpha‐synuclein (αSyn), but also contain ubiquitin and neurofilaments.^6^ Such proteinaceous inclusions occur in both familial and sporadic forms of PD, suggesting that defects in the protein handling machinery are directly related to the pathogenesis of the disease.^7^

Our current understanding of disease progression is still elusive, but considers the involvement of different disease mechanisms in different stages of the disease. Environmental factors are likely to represent triggers that start a pathological cascade of events that lead to the facilitation of molecular alterations, which are further aggravated as the disease progresses.^5^ Furthermore, mechanisms related to the regulation of gene expression have been extensively linked to the development and progression of a variety of brain diseases in recent years. For instance, alterations of miRNA expression have been linked to the development and progression of a variety of brain diseases, including PD,^8^ where deregulated miRNAs have been identified in nervous system tissues and in peripheral fluids.^9^, ^10^ Decreased levels of miR‐133b were identified in the midbrain of patients with PD and in mouse models of PD.^11^, ^12^ Alterations in the levels of miR‐34b/c were found in several regions of PD‐affected brains. These miRNAs can mimic impairments in mitochondrial function and oxidative stress, disease mechanisms believed to be crucial for the development of PD.^13^ Two miRNAs (miR‐7 and miR‐153) were also shown to regulate the expression of αSyn. Interestingly, the former has been found to be altered in the striatum and substantia nigra of patients with PD, as well as in murine models of PD.^14^, ^15^

Exploring individual profiles of the transcriptome, the microRNAome, or the proteome in PD‐affected brains is a powerful strategy to understand neurodegenerative events that underlie the disease. For instance, defects in iron metabolism,^16^ in autophagy,^17^ mitochondrial dysfunctions,^18^ and dysfunctions in synaptic function^16^, ^19^ were all captured in previous transcriptomic and/or proteomics studies. Moreover, a number of promising targets were identified by such studies and have been regarded as potential therapeutic targets or disease biomarkers for PD (e.g. NR4A2, ULK1, OR51E2, NRF2, FTL, GGH and BSCL2).^16^, ^17^, ^18^, ^19^, ^20^, ^21^ Nevertheless, singular omic profiling also encompasses a number of limitations, as it provides only a snapshot of the pathological events that might be a part of a much bigger network of deregulation, and fails to capture changes occurring up‐/down‐stream to the selected omic level (e.g. miRNA regulation or post‐translational modifications). Only a few studies have attempted to combine high‐throughput profiling techniques to explore the molecular changes that take place in postmortem PD‐affected brains, but the number of techniques employed in parallel was limited.^22^, ^23^, ^24^, ^25^ In order to present a more complete picture of the molecular events taking place in PD, here we present a comprehensive and integrative assessment of postmortem PD‐affected and control (CTR) midbrains, capturing the multi‐omic landscape of the disease, that is, genomic, miRNAomic, transcriptomic and proteomic levels. Each molecular layer was analysed individually and in an integrative fashion, aiming to depict deregulated pathways that permit the exploration of molecular alterations taking place in PD‐affected brains. Overall, our findings point to putative molecular networks involved in the pathophysiology of PD, which might improve disease monitoring and delineate novel druggable targets for this devastating disease

## 2 METHODS

### 2.1 Human postmortem midbrain samples

Human midbrain samples were provided by the Parkinson's UK Brain Bank (Imperial College London, London, England). In total, 19 PD and 12 CTR samples were obtained, shipped and processed in two different batches. Frozen midbrain tissue blocks were transported and stored under controlled temperature conditions. The samples were conceded to the Department of Neurology, University Medical Center Göttingen, Göttingen, Germany, and ethical approval was given by the Multicenter Research Ethics Committee (07/MRE09/72). Table 1 encloses a summary of clinical features about the subjects. An extended table with all clinical information available for the subjects is presented in Dataset 6 in the Supporting Information. For sampling, midbrain blocks were transferred to a cryostat chamber and kept at −20°C. Each block was punched with a 20‐G Quincke Spinal Needle (Becton Dickinson), ≈20 mg tissue was collected into reaction tubes and kept at −80°C until further use.

**TABLE 1:** Demographic and clinical characteristics of analysed patient cohorts

	**Discovery cohort**		**Replication cohort**	
	CTR	PD	CTR	PD
Number of patients	10	13	2	6
Sex (m/f)	4/6	8/5	0/2	4/2
Age at death	77.1 ± 3.4 [61–96]	78.3 ± 1.3 [71–87]	74.5 ± 5.3 [60–89]	82.7 ± 1.7 [73–93]
Disease duration (years)	NA	13.4 ± 1.3 [7–25]	NA	12.2 ± 1 [7–19]
Postmortem interval (PMI)	20.6 ± 1.2 [12–25]	14 ± 1.4 [3–24]	13 [13]	17.5 ± 1.1 [10–24]

*Notes*: Data are presented as mean ± SEM, and values in squared brackets represent the range. Statistical tests for nominal variables (sex) were performed using Fisher's exact test and for continuous variables (age and PMI) leveraged Student's *t*‐test, under a significance level of 5%. Differences between CTR and PD groups within each cohort were not significant for sex (*p*‐value = .4136 and *p*‐value = .4286, for the discovery and replication cohorts, respectively) and age at death (*p*‐value = .7893 and *p*‐value = .6731, for the discovery and replication cohorts, respectively). PMI values were significantly different in the discovery cohort (*p*‐value = .00811), but not in the replication cohort (*p*‐value = .07209). These differences were not reflected in RNA library size (Table S4) and selected deregulated proteins (Table S5).

Abbreviation: CTR, controls; PD, Parkinson's disease; PMI: postmortem interval from death until autopsy/brain sampling.

### 2.2 RNA and DNA isolation from human midbrain samples

Total RNA was isolated from human specimens using TRIzol (Invitrogen) following the manufacturer's instructions (Methods in the Supporting Information). After extraction, RNA samples were incubated at 55°C for 2 min in order to completely dissolve the RNA, and DNAse treatment (Life Technologies) was performed. RNA samples were cleaned with the RNA Clean&Concentrator‐5 Kit (Zymo Research). RNA integrity was assessed with the Agilent 6000 NanoKit in the 2100 Bioanalyzer (Agilent). DNA isolation from human midbrain samples was performed with the QIAamp DNA Mini Kit following the manufacturer's instructions.

### 2.3 RNA sequencing experiments

RNA sequencing was performed in the NIG‐NGS Integrative Genomics Core Unit, University Medical Center Göttingen. Small RNA libraries were prepared using the TruSeq Small RNA LibraryPrep Kit (Illumina) with minor modifications (Methods in the Supporting Information). The quality/integrity of RNA libraries was assessed in the Fragment Analyzer (Agilent). All sequenced samples exhibited comparable RNA integrity. Both small and total RNA sequencing were performed on the Illumina HiSeq4000 platform (Illumina), generating 50 bp single‐end reads (small RNA sequencing: 10–20 million reads/sample; total RNA sequencing: 30–40 million reads/sample). After sequencing, sequence images were transformed to binary base call files with the BaseCaller software. The files were demultiplexed to fastq files with bcl2fastq v2.17.1.14. (Illumina). Quality check was done with FastQC v.0.11.5.

### 2.4 RNA sequencing data processing and mapping

After small and total RNA sequencing, the data was processed with a customized in‐house pipeline (Methods in the Supporting Information). After adapter trimming/demultiplexing, reads were mapped to the reference genome (genome build GRCh37.p5; miRNAs/piRNAs/other non‐coding RNAs known sequences). The reads were mapped in the non‐splice‐junction‐aware mode. No mismatches for the reads < 19b were allowed. For reads between 20b and 39b, one mismatch was allowed, and for reads between 40b and 59b, two mismatches were tolerated. All other parameters were set as default in RNA‐STAR.

### 2.5 Sequential window acquisition of all theoretical mass spectra

After protein lysate preparation with Urea/Thiourea/Chaps lysis buffer (details in Methods in the Supporting Information), 50 μg of protein were loaded into a 4–12% NuPAGE Novex Bis‐Tris Minigels (Invitrogen). Following sample cleanup by electrophoresis (details in Methods in the Supporting Information), the bands stained with Coomasie Brilliant Blue (ThermoFisher) were cut out, diced, added of dithiothreitol alkylated with iodoacetamide for reduction and digested with trypsin overnight. Tryptic peptides were extracted from the gel and the solution was dried in a Speedvac. After spectral library generation (Methods in the Supporting Information), protein digests were analysed on an Eksigent nanoLC425nanoflow chromatography system (AB Sciex) hyphenated to a hybrid triple quadrupole‐time of flight mass spectrometer (TripleTOF 5600+). Qualitative liquid chromatography/tandem mass spectrometry (LC‐MS/MS) analysis was performed using a Top25 data‐dependent acquisition method (Methods in the Supporting Information). Three technical replicates per reversed‐phase fraction were analysed to construct a spectral library. During quantitative sequential window acquisition of all theoretical (SWATH) analysis, three replicate injections were acquired for each sample.

### 2.6 Mass spectrometry data processing

Protein identification was achieved using Protein Pilot Software v.5.0 build4769 (AB Sciex) at thorough settings. Spectral library generation and SWATH peak extraction were achieved in PeakView Software version 2.1 build 11041 (AB Sciex) using the SWATH quantitation microApp (v.2.0 build2003). Following retention time correction by the iRT standard, peak areas were extracted using information from the MS/MS library at a false discovery rate (FDR) of 1%.^26^ Finally, the resulting peak areas were summed to peptide area values and next to protein area values.

### 2.7 Differential expression analyses of small and total RNA sequencing data

Two complementary computational frameworks based on DESeq2^27^ were used for the differential expression (DE) analysis of small/total RNA‐seq data. Pipelines differed in the pre‐processing procedure (Methods in the Supporting Information). *p*‐values were derived by the Wald test and adjusted for multiple testing with Benjamini–Hochberg employing a 10% FDR to establish deregulated candidates. The Grubb's‐test was used to identify putative outlier samples at .05 significance level.

### 2.8 Proteomics differential expression analyses

The DE analysis of the proteomics was performed with Perseus v.1.5.6.0.^28^ This application performs multiple hypothesis testing corrections using a permutation‐based FDR approach, where Analysis of Variance and *p*‐values are computed between the measured and permuted data using Student's *t*‐test statistical hypothesis test. FDR values were calculated as fractions of accepted hits from the permuted data over the measured one. Proteins were considered statistically differentially expressed with an FDR < .1.

### 2.9 MiRNA target prediction and multi‐omics data integration

Following differential analyses, miRNAs identified by small RNA sequencing were integrated to target transcripts identified by total RNA sequencing experiments. Details about miRNAs target genes prediction are depicted in Methods in the Supporting Information. Similarly, integration between gene and protein expression data was done for genes identified in the total RNA sequencing experiments with a protein product also identified by SWATH‐MS experiments.

### 2.10 Gene ontology and pathway enrichment analyses

Gene ontology term and pathway enrichment analyses were performed using several common functional annotation databases (i.e., GO‐biological processes (BP) and cellular component (CC), KEGG (Kyoto Encyclopaedia of Genes and Genomes) and protein–protein interaction (PPI) analyses were performed in ShinyGO and STRING^29^, ^30^). Details about selected databases and scripts are depicted in Methods in the Supporting Information. Enrichment analysis was done separately for up‐/down‐regulated entities under a significance level of FDR < .1.

## 3 RESULTS

**FIGURE 1:**
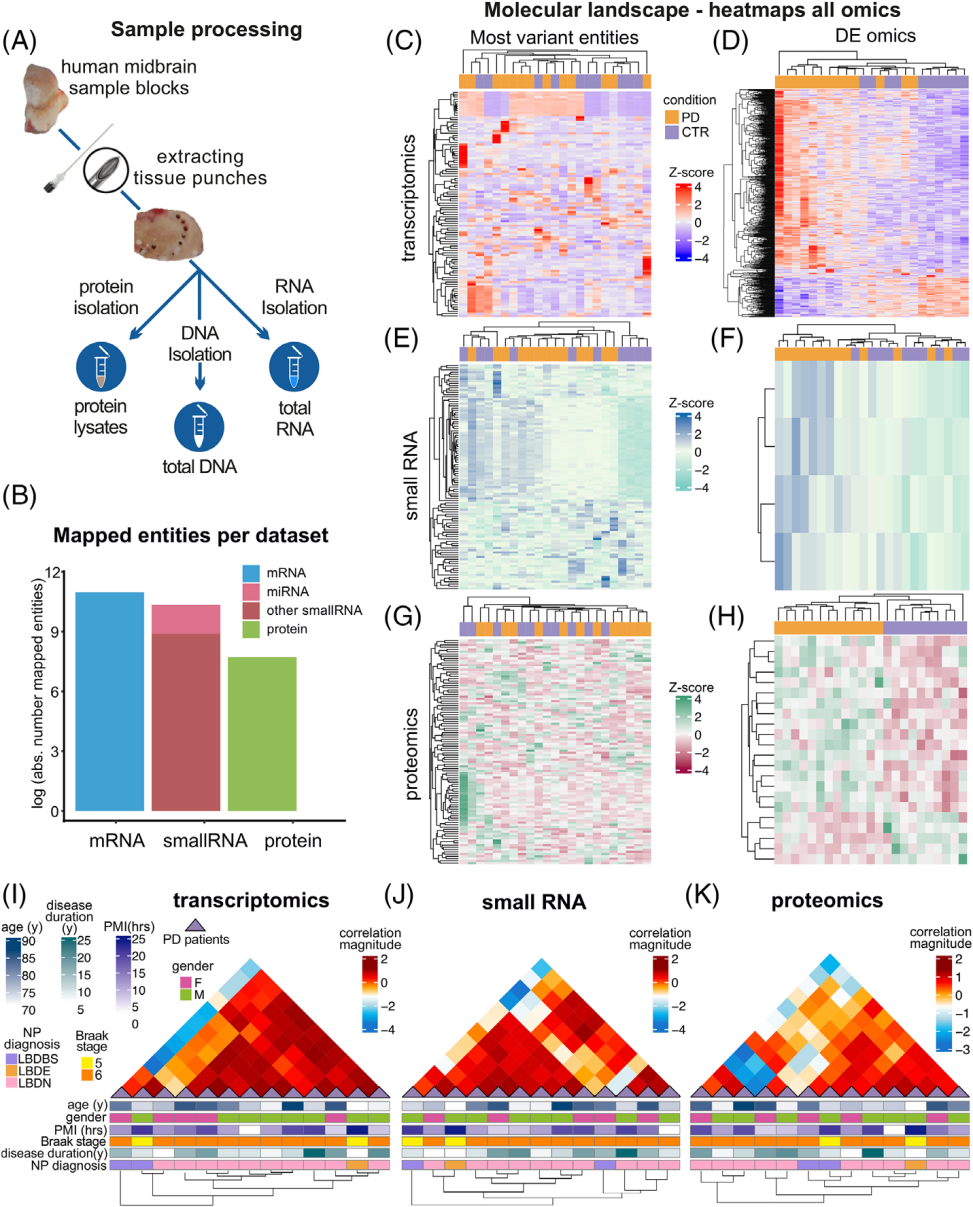
Overview of multi‐omics profiles. (A) Experimental design for the isolation of RNA, DNA and proteins from human midbrain sample blocks. The extraction of tissue biopsies was performed with a spinal needle. Adjacent tissue biopsies from each sample were used for the different isolation techniques. (B) Barplot represents the number of mapped entities for each omics dataset. The *y*‐axis represents the natural log of the mapped entities. (C–H) Heatmaps of the top 100 most variants and significantly differentially expressed transcripts, small RNAs and proteins for the discovery cohort (based on frameworks A and B, a total of 667 genes, 4 miRNAs and 22 proteins). The diagrams display the z‐score computed from the normalized counts for each individual. Column dendrograms were obtained based on the selected omics’ molecular profiles, and the row groups depict the samples' effect. Both clusters were determined using Euclidean distance and a complete hierarchical clustering. (I–K) Unbiased Bayesian hierarchical clustering of PD samples according to the total and small RNA, and proteomics expression profiles. Clinical parameters for each PD patient are represented in the lower panel of the illustrations. The column dendrograms depict the unsupervised clustering based on the correlation between patients. CTR: control; PD: Parkinson's disease; PMI: postmortem interval; Age: age at death; NP diagnosis: neuropathological diagnosis; Gender: F: females, M: males; LBDBS: Lewy body disease brainstem variant; LBDE: Lewy body disease early‐neocortical stage; LBDN: Lewy body disease neocortical stage; miRNA: microRNA

In the present analysis, we leveraged a comprehensive multi‐omics dataset of a cohort of midbrain samples including those of patients with PD and those of individuals without any indication of neurodegeneration (CTR). We obtained human midbrain tissue samples from 19 PD and 12 CTR individuals from the Parkinson's UK Brain Bank in two batches. The largest batch—comprising 13 PD and 10 CTR cases—was used as the discovery cohort and processed to obtain protein, RNA and DNA lysates, which were further subjected to a multi‐omic analysis (Figure 1A). Our discovery cohort dataset comprised 57 992 total RNA, 31 186 small RNA (of which 4383 are miRNAs) and 2257 proteins (Figure 1B). The second, independent batch of samples, 6 PD and 2 CTR cases—here referenced as replication cohort—was used for the verification of expression levels of selected candidates identified by the DE analyses in the discovery cohort.

### 3.1 Multi‐omics expression patterns and Bayesian hierarchical clustering analyses

Samples were hierarchically grouped according to the level of expression of each of the omics datasets (Figure 1C–H and Figure S6). The 100 most variable transcripts, small RNAs and proteins across the discovery cohort and the independent replication cohort resulted in mixed sample clusters between PD and CTR, indicating a high expression diversity in both groups (Figure 1C,E,G and Figure S6A–C). A Bayesian hierarchical clustering analysis of the normalized mapped counts of transcriptomics, small RNA and proteomics expression data in the PD group only showed different levels of heterogeneity with a decreasing level of positive correlation between subjects, starting from the transcriptome over the small RNA composition up to the proteome (with 64, 61 and 57 positively correlated pairs of patients for transcriptomics, small RNA and proteomics, respectively). Although sub‐clusters of expression patterns with a higher correlation magnitude were discernible, cluster compositions were not similar within multi‐omics layers. Furthermore, sub‐clusters did not correlate with patterns of respective clinical/histological parameters, suggesting that molecular diversity in PD may be independent of the clinical/histological phenotype (Figure 1I–K and Methods in the Supporting Information).

### 3.2 Genetic background of PD patients

To describe the genetic background of PD patients that were selected for this study and to exclude bias from mutations known to cause familial PD, we performed both gene panel sequencing and multiplex ligation‐dependent probe amplification (MLPA) (Figure S1). Here, the presence of mutations/duplications/triplications in genes previously associated with PD was assessed. MLPA results revealed no alterations in copy numbers in any of the analysed genes/exons (Figure S1B, Table S1). A panel of 29 genes/exons previously linked to PD or dystonia (DYT) phenotypes (Figure S1C, Table S2) was employed for targeted next‐generation sequencing analysis of the PD patient cohort. No pathogenic or likely pathogenic variants were identified using gene panel sequencing. One patient (PD6) presented a variant of uncertain significance (VUS) for the *POLG* gene (single nucleotide variant, NM_001126131.1:c. 2542G > *A*).

### 3.3 Expression profiles of small and total RNA and integrative approaches

**FIGURE 2:**
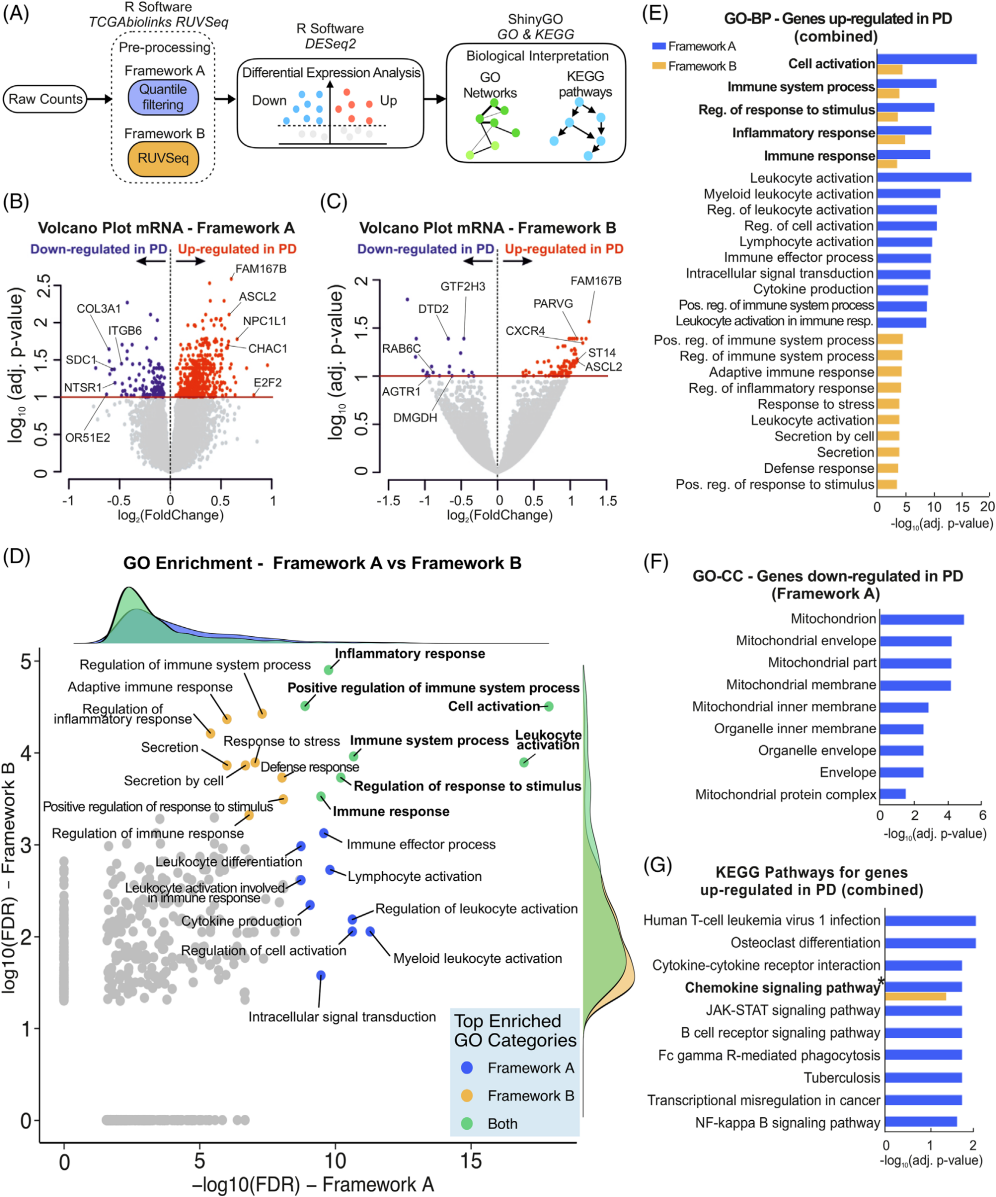
RNA‐seq data schematic workflow and analysis results for total RNA‐sequencing. (A) Illustration of the workflow of the bioinformatics pipelines used for differential expression analysis of small and total RNA sequencing data. The analyses start with the raw data expressed as integer reads for each sample and small RNA/gene. These were pre‐processed using two distinct frameworks “A” and “B” (details in Supporting Information). Then, the frameworks were further evaluated for differential expression through DESeq2^27^ and functional annotation of differential results using enrichment analysis tools available in ShinyGO.^29^ (B,C) Volcano plots portraying the differential expression of total RNA sequencing data between PD and CTR subjects, for frameworks “A” and “B”, respectively. The *x*‐axis represents log_2_(fold change) (log_2_FC) and *y*‐axis −log10(*p*‐adjusted value). Under *p*‐adjusted < .1, we found 641 and 126 differentially expressed genes for framework “A” and “B”, respectively. Genes attending these criteria are coloured in blue and red, for negative and positive log_2_FC, respectively. Highlighted genes based on the integrative analyses for RNA sequencing experiments. (D) Comparison of enriched false discovery rate (FDR) gene ontology (GO) categories obtained by frameworks “A” and “B” for the significantly up‐regulated genes (FDR < .1, yielding 500 and 427 enriched GO categories for framework “A” and “B”, respectively). Only commonly enriched categories were considered for the scatterplot. The top enriched GO categories are highlighted for framework “A” (−log10(FDR) > 8.7, a total of 16 classes, in blue), “B” (−log10(FDR) > 3.3, a total of 15 classes, in orange) and both (in green). Marginal plots represent densities of enriched GO classes for each framework and ensemble. The axis values are in the base‐10 log scale. Additionally, the GO terms not common for both frameworks were mapped to zero in the *x*‐ and *y*‐axis. (E) Top 15 GO‒*biological processes* categories enriched for genes up‐regulated in PD obtained with frameworks “A” and “B”, under FDR < .1. Bars represent log10 transformed adjusted *p*‐values. (F) GO‒cellular component categories enriched for genes down‐regulated in PD obtained with framework “A”, under FDR < .1. Bars represent log10 transformed adjusted *p*‐values. (G) Top‐10 significant KEGG signalling pathways for frameworks “A” (blue) and “B” (orange), under FDR < .1. Chemokine signalling pathway was enriched in both frameworks “A” and “B” (see Figure S14 for the full pathway). CTR: control; PD: Parkinson's disease; GO: gene ontology; BP: biological process; CC: cellular compartment; KEGG: Kyoto Encyclopedia of Genes and Genomes; FDR: false discovery rate

**FIGURE 3:**
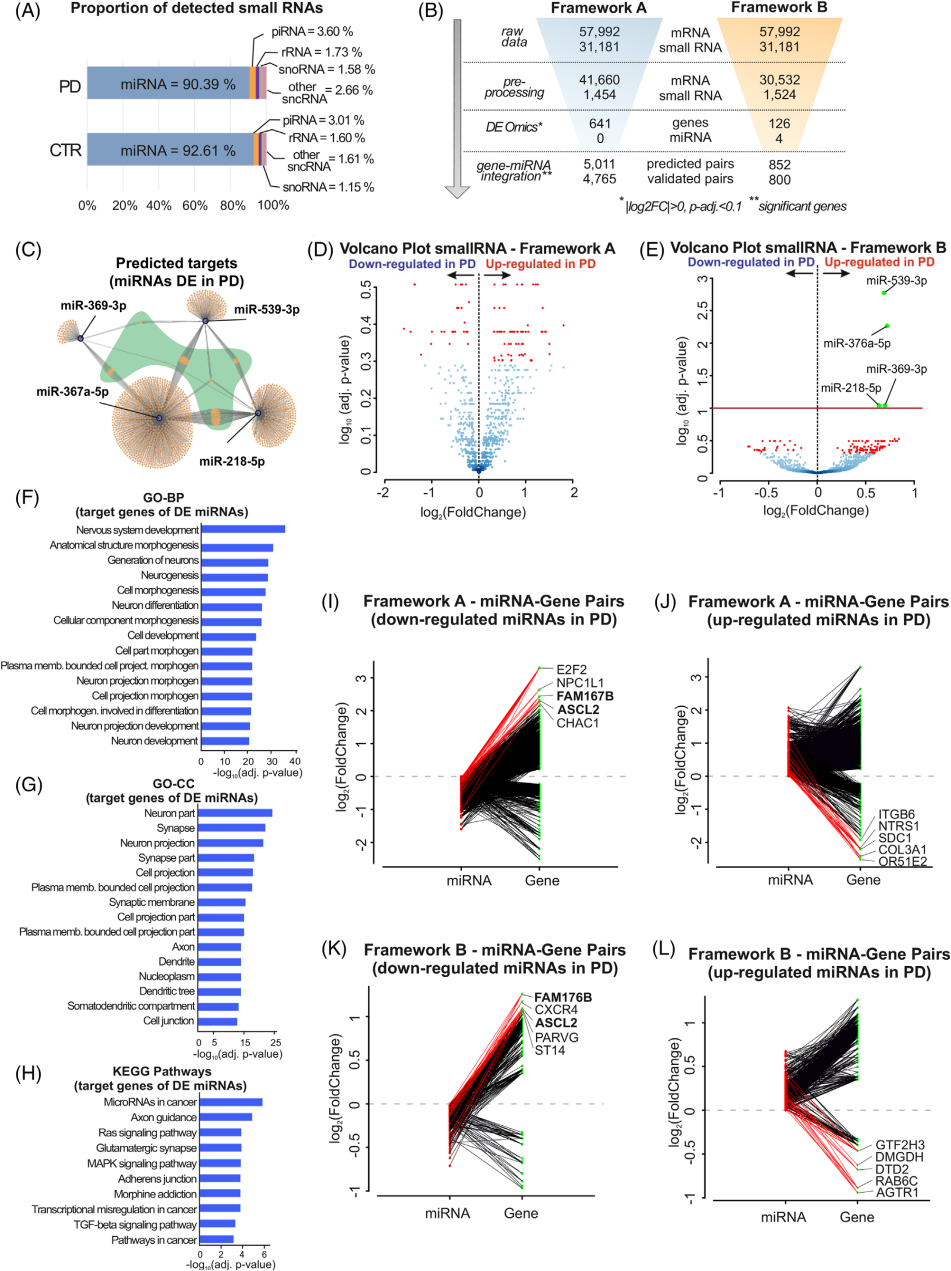
Integration of RNA sequencing experiments and analysis results of small RNA sequencing data. (A) Horizontal bars depicting the percentages of the average quantities of the different small RNA species detected in the small RNA libraries as a readout for the quality of the sequencing technique for the PD patients and CTR subjects. (B) Results obtained by frameworks “A” (blue) and “B” (orange) in each step of the differential expression and integration analyses for RNA sequencing data. (C) Predicted targets for signature‐miRNAs. Hub target genes that are common to the three miRNAs are highlighted in green. (D,E) Volcano plots portraying the differential expression of small RNA sequencing data between PD and CTR subjects, for frameworks “A” and “B”, respectively. The *x*‐axis represents log_2_(fold change) (log_2_FC) and *y*‐axis −log_10_(*p*‐adjusted value). Four up‐regulated miRNAs with framework “B” were found under *p*‐adjusted < .1. Small RNAs attending these criteria are coloured in red for positive log_2_FC. (F–H) Top 15 GO‒biological processes (GO‐BP), GO‒cellular component (GO‐CC) and KEGG Pathway terms enriched for the predicted targets of the differentially expressed miRNAs, respectively, under FDR < .1. All bars represent log_10_ transformed adjusted *p*‐values. (I–L) Differentially expressed genes obtained from frameworks “A” and “B”. All mapped microRNAs (miRNAs) were integrated with their respective validated targets (Methods in the Supporting Information). For each panel, the analysis for up‐ and down‐regulated miRNAs in PD is depicted. The *y*‐axis denotes the log_2_(fold change) of the miRNAs (in red) and genes (in green). From these pairs, we highlighted genes with a valid interacting miRNA (opposite regulation) based on their high differential level. CTR: control; PD: Parkinson's disease; DE: differentially expressed; FC: fold change; GO: gene ontology; BP: biological process; CC: cellular compartment; KEGG: Kyoto Encyclopedia of Genes and Genomes; miRNA: microRNA; piRNA: Piwi‐interacting RNA; rRNA: ribosomal RNA; snoRNA: small nucleolar RNA; sncRNA: small non‐coding RNA

After isolation of total RNA, we analysed the transcriptomic profiles and miRNA expression patterns in our cohorts using small and total RNA sequencing (Figures 2 and 3A; Figure S2A). For the DE analysis, we leveraged two bioinformatics frameworks, which we call “A” and “B” (Figure 2A). These frameworks differed in the pre‐processing stage: “A” was data‐driven and performed filtering using the distribution quantile information, while “B” took a more supervised approach and removed the negative CTR omics, that is, omics with zero effect on the variance (Methods in the Supporting Information). Following small RNA sequencing, mapping results showed that most sequencing counts were composed of miRNAs in both PD and CTR conditions (90.39% and 92.61% of all mapped counts for different small RNA species, respectively) (Figure 3B). Using the same RNA source, mRNA libraries were prepared using a strand‐specific, massive‐parallel cDNA library preparation protocol. Sequencing read counts were mapped and assigned to the reference genome, accounting for a total of 46 500 genes with valid read values before filtering.

For both frameworks, data quality assessments were conducted using unsupervised learning methods (Figures S3B–F and S4A–E). Principal component analysis and t‐distributed stochastic neighbour embedding of the small RNA seq data revealed that the control (CTR12) did not cluster with the rest of the samples (Figures S3D and S4C). The retrieved neuropathological report for this sample indicated the presence of AD‐type Tau pathology‐Braak‐stage II,^31^ and multiple small demyelinating plaques, which also suggested that it was a potential asymptomatic multiple sclerosis case.^32^ Following an outlier comparative analysis via the Grubbs’ test (*p*‐value = .033), this sample was regarded as an outlier and removed from further analyses (Figures S3E and S4D).

For all DE analyses presented here, we defined significantly different genes and miRNAs for both frameworks based on *p*‐adjusted < .1. For framework “A”, DE analyses revealed 641 deregulated genes (512 up‐ and 129 down‐regulated in PD) but no DE miRNAs (Figures 2B and 3B,D; Dataset 1 in the Supporting Information). Application of framework “B” resulted in 126 DE genes (105 up‐ and 21 down‐regulated in PD) and 4 significantly DE miRNAs (miR‐539‐3p; miR‐376a‐5p; miR‐218‐5p; miR‐369‐3p) all of which were up‐regulated in PD (Figure 2C and 3B,E; Dataset 2 in the Supporting Information). In addition, the frameworks shared 101 common genes, all with the same regulation directionality regarding PD and CTR. Our results highlight the usage of two distinct analysis pipelines for datasets encompassing multiple characteristics: framework “A” was suitable for diverse data with high variability between biological groups, such as our gene expression data (Figure S5A), whereas framework “B” was more sensitive to omic data with low expression and less dispersion, which is true for our miRNA expression data (Figure S5B).

For integrative assessment of the RNA sequencing datasets, we conducted a miRNA‐target prediction analysis of the four significantly DE miRNAs (miR‐539‐3p; miR‐376a‐5p; miR‐218‐5p; miR‐369‐3p) using databases for validated and predicted targets (Methods in the Supporting Information). We identified six predicted and four validated gene targets for these miRNAs that were also present at deregulated levels in our total RNA sequencing data (Table S6). Among the identified pairs, miR‐369‐3p/general transcription factor IIH subunit 3 (*GTF2H3)* and miR‐218‐5p/Ras‐related protein Rab‐6C (*RAB6C)* presented a discordant expression with an up‐regulation of the miRNAs and down‐regulation of transcripts in PD, representing a valid interaction between miRNAs and their target genes. Additionally, we performed an exploratory integrative analysis that considered significant genes and all of the mapped miRNAs (independent of their significance level) for both frameworks. Here, most of the regulated genes had a corresponding targeting miRNA as a valid interactor (discordant expression) (Figure 3I–L). The expression levels of the most variant miRNAs and genes with valid miRNA pairs for both discovery and replication cohorts were visualized using boxplots (Figure S16). Framework “A” yielded 4795 unique pairs of miRNAs and gene symbols (2459 with concordant levels of expression, and 2336 discordant ones), whereas framework “B” yielded 800 pairs (346 with concordant levels of expression and 454 discordant ones) (Figure 3B,I–L). Based on DE and integrative results, candidate miRNAs and genes were selected for validation by quantitative real‐time polymerase chain reaction (qRT‐PCR). This included the four miRNAs identified by framework B, which we found consistently and significantly upregulated in PD (Figure S7). In addition, we validated two of the miRNA target‐genes captured by our analyses (*RAB6C* and *GTF2H3*), and seven additional genes which were either highly deregulated or established PD pathophysiological drivers (Figure S8C). Overall, seven of those nine candidate genes presented significant regulation (*p*‐value < .05), whilst all nine genes displayed concordant gene regulation patterns in validation qRT‐PCR and RNA sequencing experiments (Figure S8A–C).

### 3.4 Verification of clinical feature effects and cell type distribution

To verify whether clinical features or cell type distribution influence the results presented above, we leveraged correlation analyses and cell type deconvolution methods.^33^, ^34^ First, we tested for correlations between clinical parameters (Dataset 6 in the Supporting Information) versus library quality results. RIN and PMI revealed no significant correlation over the whole cohort and biological subgroups (*R* = [−.2704, −.0048]; *p*‐value_cohort_ = .9826; *p*‐value_PD_ = .9389; *p*‐value_CTR_ = .4498). In addition, clinical features (i.e. RIN, PMI and disease duration) did not correlate with RNA library quality measures like % of GC content and number of total sequences (Table S4), or significantly validated proteins (Figure S9, Table S5). Consecutively, to analyse the distribution of different cell types in the human brain tissue, we used RNA sequencing decomposition methods (Methods in the Supporting Information). Data deconvolution techniques disclosed several neuronal and immune cell types that populated the analysed samples. As expected for midbrain tissue, astrocytes, neurons and oligodendrocytes were the most common cell types present (Figure S10), however no significant differences were detected in the distribution of cell types in PD or CTR. Furthermore, a specific deconvolution for immune cells was performed and indicated infiltration/proliferation or increased differentiation towards the granulocyte/monocyte lineage in the analysed midbrains (Table S3). Finally, we investigated the presence of sex‐related effects with DE analyses using “framework A” (Methods, Figure S11, Dataset 4 in the Supporting Information), which revealed no significant differences in the composition of the discovery cohort in terms of sex (*p*‐value = .4136; Table 1). Sex‐regressed results greatly overlapped with the analysis without sex adjustment: 81% of all genes identified in the main analyses remain significant after controlling for sex, and 20 out of 23 candidates underlined by the integrative analyses in our study were significantly expressed in both approaches. No pathways were enriched for the genes identified strictly when correcting for sex. In addition, the majority of sex‐specific genes were not significantly deregulated in both approaches (Figure S11, Dataset 4 in the Supporting Information). Based on the correlation studies with clinical parameters, gender information and cell decomposition analyses, no clinical features or cell proportions were considered as covariates in the DE analysis presented here.

### 3.5 Functional annotation for RNA sequencing results and weighted correlation network analysis

Functional annotation of the DE genes in PD revealed several biological processes that were shared between frameworks that have been known or suspected to be relevant to the pathogenesis of PD (Figure 2D–G). Most of these were related to immune and inflammatory responses (53% and 42% for each framework, respectively) (Figure S12). Subsequently, terms related to response to stress/apoptosis and metabolic/biosynthetic processes characterized the DE genes obtained with framework “A”, each accounting for 5% of all enriched terms (Figure S12A). In contrast, framework “B” identified differentiation and development and cytoskeleton organization (8% and 6% of the enriched categories, respectively) (Figure S12B). KEGG pathway analysis^35^ showed enrichment for several pathways involved in infectious diseases (e.g. HTLV‐1 infection, tuberculosis), several immune‐related pathways (cytokine‐cytokine receptor interaction, B‐cell receptor signalling pathway), but also cancer‐related processes (transcriptional misregulation in cancer, JAK‐STAT and, NF‐kappa B signalling) for the up‐regulated genes from framework “A” (Figure 2G). One commonly enriched KEGG pathway, chemokine signalling pathway, was identified for both frameworks. For down‐regulated genes, which were considerably fewer than the up‐regulated ones, no GO‐BP term reached significance (FDR < .1). Nevertheless, GO‐CC enrichment revealed several mitochondria‐related processes for down‐regulated genes obtained with framework “A” (Figure 2F).

In order to explore the biological role of the four deregulated miRNAs obtained using framework “B”, we performed functional enrichment analyses with their predicted target genes (Figure 3C). Neuron‐related pathways were the most common among enriched GO categories (Figure 3F–H): neuron system development and neurogenesis related pathways were the most significant GO‐BP terms, while neuron part, synapse and neuron projection were the most enriched GO‐CC annotations. Moreover, KEGG pathways related to axon guidance and glutamatergic synapse were also highly significant for the target genes of deregulated miRNAs. A network visualization (Figure S15) disclosed eminently enriched KEGG pathways with several shared genes, for example, MAPK and Ras signalling pathways.

In addition to functional annotation, we performed a weighted correlation network analysis (WGCNA) of the discovery cohort gene counts with a power parameter of 14 (Methods in the Supporting Information). This analysis yielded 130 gene modules, from which eight were selected for further analysis based on their significance between biological conditions (p‐value < .05; Dataset 5 in the Supporting Information). WGCNA results were also integrated with significantly DE miRNAs (miR‐539‐3p; miR‐376a‐5p; miR‐218‐5p; miR‐369‐3p) through validated targets dataset (Figure 5). Pathway analysis for the gene content of these modules (Figure 5; Figure S13) revealed similar pathways as previously seen in GO and KEGG analyses. These included modules strongly enriched for terms as nervous system development and neuronal/glial differentiation (Module ME1), inflammatory and immune response (Modules ME28 /ME30), transcriptional regulation (Module ME11), cell metabolism and chromatin organization (Module ME26).

### 3.6 Protein expression profiling, functional annotation, integration with RNA sequencing data

In order to profile the proteomics changes in our samples, we subjected midbrain tissues of our discovery cohort to SWATH‐MS (Figure S2B). Following preparation of an annotated peptide spectral library, we were able to detect and quantitate a total of 2257 proteins across all samples at 1% FDR.

**FIGURE 4:**
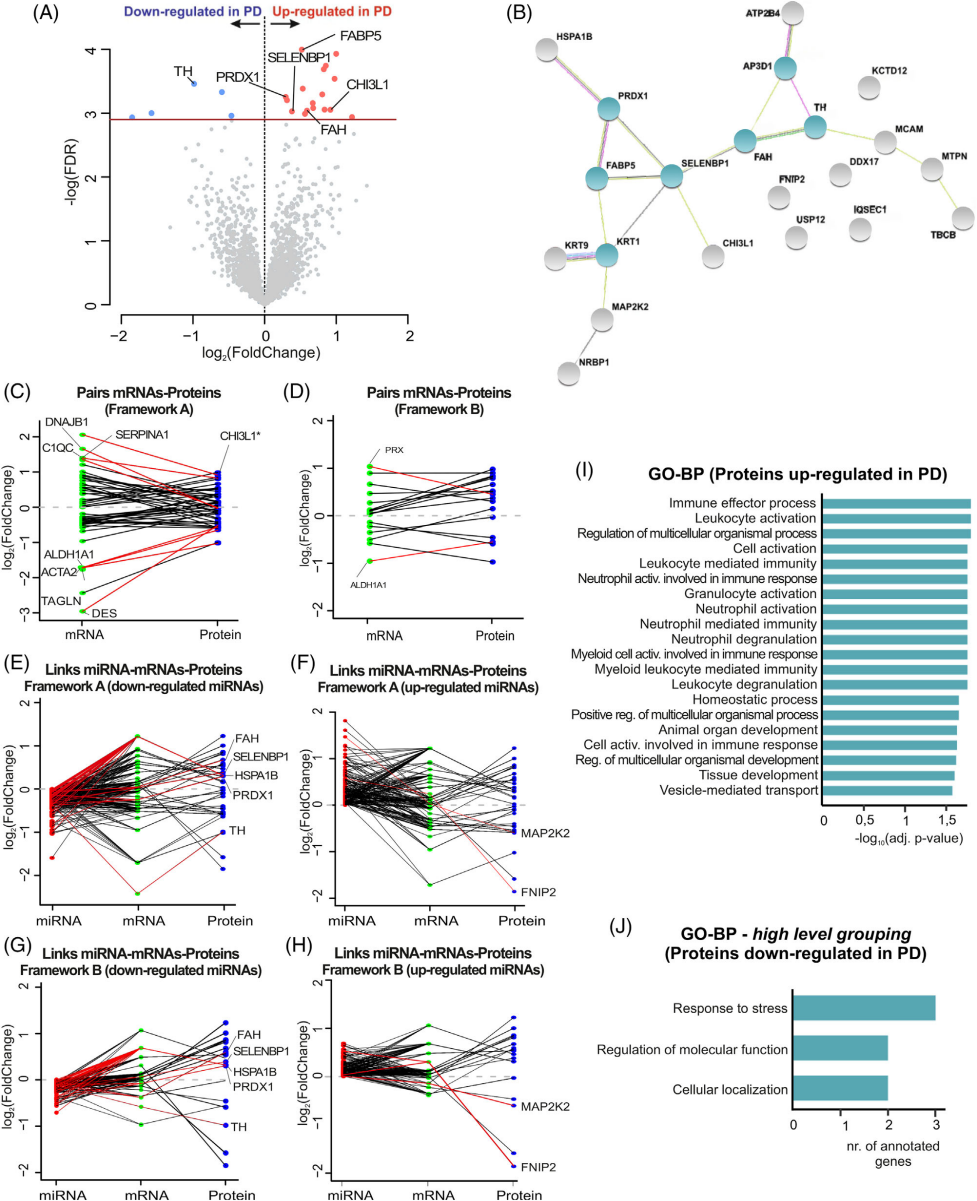
Proteomics analyses of midbrain samples. (A) Volcano plot showing all detected proteins in midbrain samples of PD and CTR subjects. Differentially expressed proteins (total of 22) between CTR and PD indicated in blue (down‐regulated in PD) and in red (up‐regulated in PD). Horizontal line depicts the cut‐off for significance (FDR = .1). (B) STRING analysis for the differentially expressed proteins. Clusters were defined by the Markov Algorithm in STRING 11.0,^30^ using default settings. Hub proteins (3 or more links) are highlighted in green. (C,D) Differentially expressed genes obtained from frameworks “A” and “B” (*p*‐adjusted < .1) and genes whose corresponding protein was significantly deregulated in the same direction (FDR < .1; 11 and 13 genes in frameworks A and B, respectively), integrated with all mapped proteins. The *y*‐axis denotes the log_2_(fold change) of the genes (in green) and proteins (in purple). From these pairs, genes are highlighted based on their high differential level (top four genes with largest differential level, |log_2_FC| > 1.4). (*) highlights CHI3L1, a candidate identified significantly up‐regulated in both transcriptomics and proteomics datasets. (E–H) Combination of the resulting pairs of small RNAs and their respective target genes from frameworks “A” and “B” (independently of their significance), with the 22 significantly expressed proteins and proteins whose corresponding gene was significantly deregulated in the same direction (*p*‐adjusted < .1; 18 and 3 proteins in frameworks A and B, respectively). The *y*‐axis denotes the log_2_(fold change) of the miRNAs (in red), genes (in green) and proteins (in purple). (I,J) Enriched GO‒biological process categories for up‐ and down‐regulated proteins in PD, respectively. CTR: control; PD: Parkinson's disease; DE: differentially expressed; FC: fold change; GO: gene ontology; BP: biological process; CC: cellular compartment; miRNA: microRNA

DE analyses after normalization for the discovery cohort revealed 22 significantly deregulated proteins between the PD and CTR groups. In the PD group, 17 proteins were up‐regulated while five proteins were down‐regulated (Figure 4A; Dataset 3). Analysis of PPI networks including all significantly deregulated proteins revealed several hub proteins and interactors, including TH, selenium‐binding protein 1 (SELENBP1), fumarylacetoacetase (FAH), fatty acid‐binding protein 5 (FABP5), HSPA1B, MAP2K2, FNIP2 and peroxiredoxin‐1 (PRDX1)(Figure 4B). The significantly deregulated proteins were matched to the transcriptomics results (Figure 4C,D), and the four most deregulated genes (up‐ and down‐regulated in PD) with their respective significant protein products were highlighted (up‐regulated: chitinase‐3‐like‐protein 1 (CHI3L1), DNAJB1, C1QC and SERPINA1; down‐regulated: ALDH1A1, ACTA2, TAGLN and DES; (|log_2_FC| > 1.4). Only CHI3L1 appeared significantly up‐regulated in the PD group, in both proteomics and total RNA sequencing. Similar to the integration of transcripts and miRNA, all mapped proteins were now related to the transcriptomic analysis and miRNA data, and this revealed several links with previous datasets (Figure 4E–H). Functional enrichment of proteins up‐regulated in PD showed the involvement of pathways related to inflammatory and immune responses (12 of the top 20 most significantly enriched categories) (Figure 4I). Despite the small number of down‐regulated proteins in the PD group, high‐level GO term grouping yielded a functional enrichment to the category's responses to stress, cellular localization and regulation of molecular function (Figure 4J). Consecutively, the expression of selected candidates was verified in an independent replication cohort (Figures S16 and S17).

Finally, protein candidates were selected for validation by Western Blotting based on proteomics DE results and integrative approaches within multi‐omic datasets. Six proteins were selected based both on the deregulation shown in proteomics experiments and on the relevance to PD pathology (namely, CHI3L1, HSPA1B, USP12, TH, FNIP2 and ALDH1A1). The down‐regulation of TH and FNIP2 was confirmed by Western Blotting (Figure S9E–G). In addition, we also validated the up‐regulation of CHI3L1 and HSPA1B (Figure S9B–D). We observed concordant trends that ALDH1A1 levels were lower and USP12 levels were increased in PD patients compared to CTR subjects, on average (although not statistically significant; *p*‐value_ALDH1A1 _= .09; *p*‐value_USP12 _= .5) (Figure S9B–E).

## 4 DISCUSSION

The lack of disease‐modifying therapies in PD is a constant reminder of the need to better understand the molecular mechanisms underlying its pathology. A better characterization of the molecular changes in PD could allow more effective/etiology‐driven therapies for this neurodegenerative disorder. Profiling the molecular landscape of PD‐affected brains may reveal pathological events taking place in the course of the disease, both at the cellular and systemic levels, pinpointing deregulated molecular pathways and novel druggable targets. In this study, we leveraged the potential of multi‐omic tissue analysis to characterize pathology‐related molecular alterations in postmortem midbrain tissue of PD patients and age‐correlated controls.

As our analysis aimed to identify pathways that characterize sporadic PD, we first excluded the presence of genetic alterations (known to cause monogenic forms of PD) in our samples. Comprehensive mutational screening using gene panel sequencing and by MLPA indicated the absence of genetic abnormalities for known PD genes in the discovery PD cohort (Figure S1, Table S1). A single‐nucleotide VUS in the *POLG* gene was identified in one PD patient who belonged to the independent replication cohort; therefore, it was not included in the main analysis. Similar nucleotide variances in the *POLG* gene have been linked to alterations in PD predisposition in Finnish and Chinese populations, but mechanistic evidence of its contribution to the pathophysiology of PD is lacking.^36^, ^37^ Our findings confirmed that the discovery cohort is composed of idiopathic PD cases and excludes a major influence of genetic alterations in our profiling results. In addition, we found no significant differences in the cohort composition in terms of sex (Table 1), and no significant influence when controlling for sex in the DE analysis (Figure S11). Most importantly, almost all of the candidates that we identified by our integrative analyses (20 out of 23) were present when correcting or not for sex, while most of the sex‐specific genes did not present significant regulation in the analysed cohort (Figure S11). Overall, these findings confirm that sex differences did not exert major influences in the analyses presented here.

Next, unbiased hierarchical clustering analyses demonstrated a high heterogeneity and did not permit clustering of subjects according to disease entity in the top most variant samples (Figure 1C,E,G; Figure S6A–C) and identified DE transcripts and small RNAs (Figure 1D,F; Figure S6D–F). In contrast, DE proteins revealed a strong clustering of biological subgroups in the discovery cohort (Figure 1H). In the clustering analysis of the PD cohort, the transcriptomics analysis showed the highest homogeneity among subjects, whereas small RNAs and proteins were more heterogeneous (Figure 1I–K), arguing for changes induced by post‐translational modifications. The number of post‐mortem samples in this project was too small to identify distinct patient subgroups; however, we recently analysed the diversity of miRNA in cerebrospinal fluid (CSF) of patients with PD, which revealed distinct molecular subgroups that were independent of the clinical phenotype.^38^ A future analysis of a larger number of samples could be more suited to identify distinct subgroups and will represent an important prerequisite for the development of personalized therapeutic approaches based on the molecular phenotype in PD.

DE analysis of RNA sequencing data was conducted using two different frameworks for the pre‐processing stage (Figure 2A), each one having a different focus, for a broader and more complete investigation of different RNA sequencing datasets with distinct count distributions (Figure S5). While framework “A” considered a distribution‐driven approach appropriate for sparse data, framework “B” had a variance‐based supervised procedure. A considerable number of genes were found to be significantly deregulated in framework “A”, while no statistically significant miRNAs could be detected. In contrast, application of framework “B” resulted in multiple deregulated genes and miRNAs (Figure 2D). Analysis of the RNA sequencing data using two different frameworks provided a holistic view of the data sets and revealed subtle levels of deregulation that might not have been captured if only one or the other system had been used. Because our data spanned multiple omics layers, we examined all deregulated candidates (independent of log_2_FC) for integration analysis and then focused on the top hits for individual omics analyses (results with other log_2_FC thresholds can be found in Table S7). Overall, this depicts the importance of choosing the most suitable bioinformatic paradigm covering the variety of human expression data (Figure S5).

Differential transcriptome analyses revealed that there are more up‐ than down‐regulated genes in PD (Figure 2B,C), which is in line with a recent meta‐analysis on substantia nigra transcriptome.^39^ Transcriptomic overexpression was on average more common in the PD groups both at the single gene level and also when analysing chromosomal segments with expressed sequencing tags. Interestingly, despite the different transcripts obtained with each framework, both the regulation directionality and the enrichment results were very similar in both settings. Up‐regulated genes in PD were largely enriched to inflammatory and immune responses, but also to terms related to stress and apoptotic responses, cytoskeleton organization, differentiation and development and metabolic‐related processes (Figure 2E; Figure S12A,B). Processes related to immune cell proliferation and defence response were more enriched in framework “A” than they were in “B”, which might be due to the smaller number of significantly deregulated genes yielded by the latter (Figure 2D). Similarly, KEGG pathway enrichment results are related either to inflammation/immune responses, or to a variety of infectious diseases (Figure 2G), likely because pathways of infectious diseases contain genes related to immune cell activation and inflammation. One particular pathway—chemokine signalling pathway*—*which is directly related to inflammation/leukocyte recruitment,^40^ was shared by both frameworks (Figure 2G; Figure S12).

Moreover, transcriptomics analysis through framework “A” also yielded functional enrichment for stress/apoptosis and metabolic/biosynthetic processes (Figure S12A,B), reflecting features of neurodegeneration occurring in PD‐affected midbrains.^2^ Metabolic dysfunction has been frequently linked to the pathogenesis of PD^41^ and dopaminergic neurons are known to be especially sensitive to oxidative stress and mitochondrial dysfunction.^42^ Strikingly, PD down‐regulated genes also showed enrichment for numerous mitochondrial processes, known to be markedly impaired in autosomal recessive forms of PD^43^ (Figure 2F). Framework “B” led to enrichment for differentiation and development, and a similar enrichment was observed for the deregulated miRNAs. Cell cycle‐related proteins play an important role in the survival of mature neurons, and also in neuronal apoptotic processes.^44^ Overall, those findings support the connection between the miRNA and gene expression datasets, encouraging the integrative efforts employed in this study.

**FIGURE 5:**
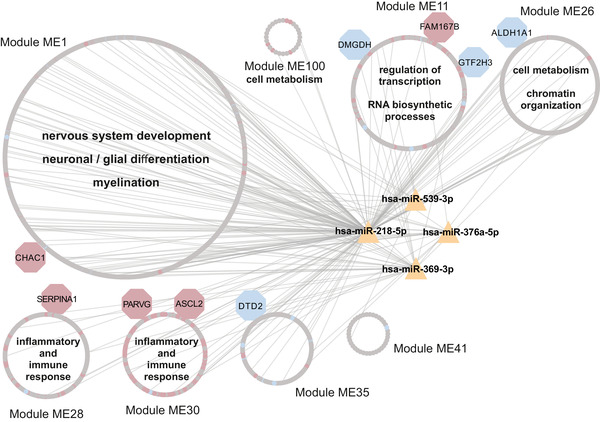
Visualization of top significant WGCNA gene modules and DE miRNAs. WGCNA analysis revealed 8 significant gene modules (*p*‐value < .05) between PD and CTR. Through the validated miRNA targets database, the network was extended to the significantly DE miRNAs (in yellow) and edges between genes and miRNAs were constructed. The red and blue nodes represent significantly up‐ and down‐regulated genes (*p*‐adjusted < .1) in frameworks “A” and “B”, respectively. Grey nodes represent genes in WGCNA modules with no significance (*p*‐adjusted > .1). Gene nodes of bigger size (DMGDH, FAM167B, GTF2H3, ALDH1A1, CHAC1, SERPINA1, PARVG, ASCL2 and DTD2) are highlighted as relevant gene candidates due to their significant differential expression and integration with miRNAs and proteins (see Figures 2, 3, 4). Furthermore, gene ontology analysis of genes in each WGCNA module revealed several significant biological processes (FDR < .1; Figure S10), which were summarized and represented in each of the illustrated WGCNA modules (terms depicted inside module circles). Modules with high distribution of up‐regulated genes (e.g. ME28 and ME30) translated high significance in inflammatory and immune response pathways. The remaining WGCNA modules and genes in these modules (*p*‐value > .05) can be found in the Dataset 5 in the Supporting Information. CTR: control; PD: Parkinson's disease; DE: differentially expressed; miRNA: microRNA; WGCNA: weighted correlation network analysis

For the small RNA data, we were able to identify four differentially expressed miRNAs using framework “B”, all of which were up‐regulated in PD (Figure 3G). Similar findings also suggested an overall up‐regulation of circulating miRNAs in PD.^45^, ^46^ Remarkably, all candidates depicted here have already been linked to PD and other neurodegenerative diseases. miR‐218‐5p was found up‐regulated in peripheral blood mononuclear cells (PBMCs) of PD patients and its levels were reversed after deep brain stimulation therapy.^47^ In the CSF of Alzheimer's disease (AD) patients, miR‐218‐5p was found to be decreased.^48^ miR‐376a‐5p was found increased both in PD PBMCs and in vitro. Interestingly, it regulates genes that are involved in mitochondrial dysfunction and were previously linked to PD pathogenesis (*TFAM; PGC1α*),^46^ but also *SIRT2*,^49^ a protein implicated in PD for its effects in α‐syn aggregation.^50^ It has also been postulated as a biomarker for PD, since its levels in PD PBMCs also seem to correlate with disease severity.^51^ One study showed elevated levels of miR‐369‐3p in PD postmortem substantia nigra,^52^ while alterations in the striatal levels of miR‐539‐3p were reported in a rodent model of PD.^53^ Functionally, these miRNAs were linked to neuron‐related gene ontology terms, indicating the neuronal origin of these miRNAs (Figure 3J–L). Additionally, terms related to cell‐cycle, proliferation, differentiation and development were highly enriched. These are known to also play a vital role in the survival and maintenance of mature neurons^54^ and are directly influenced by miRNA levels during neurodegeneration.^53^ In concordance with the DE analyses, WGCNA results also revealed a strong enrichment of up‐regulated genes in inflammation‐related modules (Figure 5), as well as modules related to nervous system development, cell metabolism and transcriptional regulation (Figure 5 and Figure S13). Highlighting the importance of these pathways, the results of the WGCNA also underscore the role of the four deregulated miRNAs identified in the DE analysis.

To obtain a more holistic view, the RNA sequencing datasets comprising transcriptome and microRNAome were analysed in an integrative fashion (Figure 3E–I). PD up‐regulated genes included transcription factor *E2F2*, implicated in hemisphere‐dependent PD pathology^55^ and neuroinflammation after spinal cord injury,^56^ Glutathione‐specific gamma‐glutamylcyclotransferase‐1 (*CHAC1*), reported to regulate unfolded protein response^57^ and linked to paraquat‐induced neurotoxicity,^58^ and *CXCR4*, a chemokine receptor linked to neuroinflammation which is expressed in dopaminergic neurons.^59^ The latter was experimentally validated and showed strong up‐regulation in the PD samples analysed here (Figure S8B). *DRD2* and *OR51E2* presented marked down‐regulation in PD. The former has been found in altered levels in PD substantia nigra and has been regarded as a susceptibility locus for PD,^39^, ^60^ whereas the latter is postulated as a therapeutic target for PD. It is involved in neuromelanin pigmentation in dopaminergic neurons and found down‐regulated in PD‐affected brains.^21^, ^61^

When considering only significantly differentially expressed miRNAs and transcripts, the list was reduced to two valid miRNA‐target gene pairs: miR‐218‐5p/*RAB6C* and miR‐369‐3p/*GTF2H3* (Table S4). Importantly, we have validated the expression of those four candidates by qRT‐PCR, adding further evidence to the relevance of these pairs for the pathophysiology of the analysed PD brains. The *RAB6C* is a member of Rab GTPases, which are pivotal regulators of intracellular protein transport and vesicle trafficking.^62^ RAB6C has been implicated in autophagy and proteostasis and increased levels of Rab6 isoforms have been linked to protein aggregation in vitro^63^ Importantly, Rab GTPases are substrates of *LRRK2*, the most commonly mutated gene in familial cases of PD. Disruption of Rab phosphorylation in the *LRRK2* site leads to neurotoxicity in vitro and dopaminergic neurodegeneration in vitro.^64^ The *GTF2H3* is directly involved in RNA transcription and nucleotide excision repair.^65^ Although *GTF2H3* has not been directly implicated in PD, dysfunctions in nucleotide excision repair play an important role in chronic neurodegenerative disorders.^66^ Moreover, members of its family (TFIIH) have been shown to play a role in oxidative stress and mitochondrial DNA alterations in PD.^67^ Altogether, these candidates underline pathways that are involved in the pathogenesis of PD and support the involvement of gene expression regulation by miRNAs as a disease mechanism. The regulation of these targets is supported by the independent replication cohort (Figure S9), and the experimental validations of selected genes (Figures S7 and S8).

Finally, proteomic profiling ensured a more complete characterization of disease‐relevant post‐translational mechanisms. In line with transcriptomics, most differentially regulated proteins were increased in PD (Figure 4A). As expected, we found a marked down‐regulation in tyrosine hydroxylase (TH), the rate‐limiting enzyme for dopamine synthesis and a hallmark of dopaminergic neuron depletion in PD.^2^, ^68^ PPI networks revealed other important hub‐proteins (Figure 4B), such as the SELENBP1, FAH, FABP5 and PRDX1. Remarkably, we previously identified selenium as part of a bioelement‐signature in the CSF that allowed for differentiation between PD and CTR samples.^69^ FAH and PRDX1 were reported to play a role in oxidative stress damage in PD, and the latter has also been postulated as a new therapeutic target for PD because of its role in the generation of reactive oxygen species.^70^, ^71^ FABP5 has been linked to postnatal neurogenesis,^72^ and also to neuronal oxidative damage in vitro.^20^ Another important PD up‐regulated protein that was experimentally validated was heat shock 70 kDa protein 1B (HSPA1B), a chaperone known for its role in protein folding and degradation as well as neuronal apoptosis (Figure S9B,C)^73^. Chaperones are known to interact with αSyn,^74^ and defects in that system contribute to αSyn misfolding and the formation of inclusions/protein aggregates in PD.^75^

Significantly deregulated proteins were substantially fewer than deregulated transcripts, most likely because only the most abundant/uniquely identified proteins within all samples were considered for quantification. Furthermore, proteomics and RNA sequencing results are fundamentally very different, starting with the nature of the signals and the coverage of those techniques limiting the overlap between such datasets.^76^ However, their integration resulted in a better understanding of the regulation of identified candidate molecules, for example by comparison of individual protein expression levels to respective RNA sequencing results (Figure 4C,D) and by analysis of expression levels in the independent replication cohort (Figure S16).

An important finding was the identification of CHI3L1 which was up‐regulated in both transcriptomics and proteomics datasets, and was validated by both qRT‐PCR and Western Blot. It also showed the highest levels of up‐regulation in both analyses after integration (Figure 4C). CHI3L1, also known as YKL‐40, is widely expressed in immune cells and regulates inflammatory responses, tissue injury and repair.^77^ Remarkably, it has been previously identified in CSF studies as a potential circulating biomarker for several neurodegenerative diseases, including AD, amyotrophic lateral sclerosis and PD.^78^, ^79^, ^80^ It was also considered as a pivotal marker for immune/inflammatory changes in tauopathies.^81^ Functional analysis highlighted the GO‐BP immune effector process as the most enriched term for the up‐regulated proteins in PD (Figure 4I), matching the massive immune/inflammatory activation reported for RNA sequencing results and the deregulation of several immune mediators in the proteomics dataset. Remarkably, functional alterations related to neuroinflammation/immune response activation were captured across all omics results presented in our study and were independent of data handling paradigms. Neuroinflammation has already been considered to be a predictive feature for the appearance of non‐motor symptoms and cognitive decline in patients with PD,^82^ and inflammation‐related mechanisms seem to accompany the pathophysiological events of PD even from earlier stages.^5^, ^83^ A recent study showed that neuroinflammatory mechanisms are triggered with the release of αSyn from apoptotic neurons, aggravating the disease through a number of pathways that include microglial activation, mitochondrial damage and inflammasome formation.^84^ Overall, the integrative results from diverse omics layers depicted in this study provide further evidence for the role of neuroinflammation in PD pathology and suggest its exploitation as a therapeutic target.

It is important to stress that factors modulating protein metabolism (e.g., synthesis and degradation rates of proteins) were out of the scope of this study and were therefore not considered for the integrative analyses presented here, although they likely contribute to the overall picture. The analysis of the phospho‐proteome and the metabolome was also not included because of the postmortem nature of the source material. Limitations also apply to the number of analysed subjects and the nature of the material analysed, because high‐quality post‐mortem tissues with excellent clinical characterization are scarce, particularly for CTR subjects without neurodegenerative pathology. Nevertheless, we demonstrated that quality CTR measures, such as RIN and PMI, showed no correlation to data quality (Table S4), similar to what has been reported previously.^85^, ^86^, ^87^ Furthermore, clinical parameters in PD had no major effect on the differential results (Table S5), and there was no difference in cell type composition between samples (Figure S10), excluding a major effect of these factors on the results. While information about medication for the analysed PD cohort was incomplete, the clinical data presented here does not indicate the effects of medication on disease progression. Moreover, Levodopa—the most common symptomatic antiparkinsonian treatment—has been shown to not alter the course of PD in terms of progression.^88^, ^89^

A caveat of our data is that we analyse a snapshot of advanced PD stages, that is, patients with a disease duration of 7–25 years. As all analyses strongly underscored the role of neuroinflammation, we do not expect that an increase in sample number would change this overall picture. Nevertheless, an analysis of larger cohorts will be much more powerful for subgroup identification and may identify additional molecular targets characterizing subtypes of PD pathology.

## 5 CONCLUSIONS

In summary, our integrative multi‐omics analyses identified multiple levels of deregulation in human midbrains affected by PD, several of them overlapping across the different datasets. We identified and experimentally validated four deregulated miRNAs: miR‐539‐3p, miR‐376a‐5p, miR‐218‐5p and miR‐369‐3p. These miRNAs contribute to the regulation of transcription and inflammatory pathways, highlighting these mechanisms in the context of PD pathology. We also identified and validated two miRNA‐target gene interacting pairs (miR‐218‐5p/*RAB6C* and miR‐369‐3p/*GTF2H3*). The deregulation of these players underlines the involvement of vesicle trafficking, proteostasis and oxidative stress in the pathogenesis of PD. Established PD‐related proteins, such as TH, were also captured in our study, along with less evident candidates, such as CHI3L1, FNIP2 and HSPA1B.

The integrative view of diverse multi‐omics layers confirmed an important enrichment of neuroinflammation‐related molecules, underscoring the pivotal role for neuroinflammation in the pathogenesis of advanced PD and delineating a yet unaddressed drug target in this disease stage.^90^ Further mechanistic and clinical studies would be required to substantiate the pathological importance of our findings in the context of earlier disease stages.

## CONFLICT OF INTERESTS

The authors declare no conflict of interest.

## Supporting information

Supplementary Dataset 1—Differential results RNA sequencing Framework A plus interacting pairs (.xls)

Supplementary Dataset 2—Differential results RNA sequencing Framework B plus interacting pairs (.xls)

Supplementary Dataset 3—Differential Results Proteomics (.xls)

Supplementary Dataset 4—Comparison between regressed and non‐regressed results (for covariate sex ‐ transcriptomics) and functional annotation results (.xls)

Supplementary Dataset 5—WGCNA analysis results: modules, genes and respective differential results of Frameworks A and B (.xls)

Supplementary Dataset 6—Extended clinical information for all subjects.

Supporting Information
