# Supplementary material for: Multi‐omic landscaping of human midbrains identifies disease‐relevant molecular targets and pathways in advanced‐stage Parkinson's disease
Source: Clin Transl Med. 2022 Jan 28;12(1):e692. doi: 10.1002/ctm2.692 (PMC8797064; doi:10.1002/ctm2.692)
Supplement: Supplementary file 7 — Supporting Information [file CTM2-12-e692-s007.docx]

Supporting Information for

**Multi-omic landscaping of human midbrains identifies disease-relevant molecular targets and pathways in advanced-stage Parkinson’s disease**

Lucas Caldi Gomes^1,2,*^, Ana Galhoz^3,4,*^, Gaurav Jain^5^, Anna-Elisa Roser^2^, Fabian Maass^2^, Eleonora Carboni^2^, Elisabeth Barski^2^, Christof Lenz^6,6a^, Katja Lohmann^7^, Christine Klein^7^, Mathias Bähr^2,5^, André Fischer^5,8^, Michael P. Menden^3,4,9,§^, Paul Lingor^1,10,§#^

***Corresponding Author:** Paul Lingor; **email:** [paul.lingor@tum.de](mailto:paul.lingor@tum.de); Department of Neurology, School of Medicine, University Hospital rechts der Isar, Technical University of Munich, Ismaninger Straße 22, 81675 Munich, Germany, Tel.: +498941408257

§,* These authors contributed equally.

**Supporting Information – Table of Contents**

Supplementary Methods

Supplementary Figures

Supplementary Tables

Supplementary References

**Supplementary Methods**

**Sampling human postmortem midbrain tissue blocks**

Frozen midbrain tissue blocks of 19 PD and 12 CTR samples were provided by the Parkinson’s UK Brain Bank (Imperial College London, London, England), in two different batches. The tissue samples were transported and stored under controlled temperature conditions. Table 1 encloses all clinical information about the subjects. For sampling, midbrain blocks were transferred to a cryostat chamber and conducted at -20°C. Each block was punched 3-4 times at a depth of 5mm with a 20-G Quincke Spinal Needle (Becton Dickinson). Whenever areas of pigmentation were visible, we took biopsies directly from these. ~20 mg tissue was collected into reaction tubes and kept at -80°C until further use. A schematic of the sampling is shown in **Fig.1A**.

**DNA Isolation**

DNA isolation from human midbrain samples was performed with the QIAamp DNA Mini Kit following the manufacturer’s instructions. Nucleic acid concentration and purity were measured in the NanoDrop One spectrophotometer (ThermoFisher).

**Multiplex Ligation-dependent Probe Amplification (MLPA)**

MLPA experiments were performed in order to detect abnormalities in copy numbers (e.g. deletions, duplications, triplications) of specific PD-related genes using a set of standard commercial probes (SALSA P051 and P052 Parkinson MLPA kits, MRC Holland, Amsterdam, The Netherlands). MLPA experiments were conducted according to the manufacturer's protocol. PCR amplification products were visualized on capillary sequencing machines (either ABI 3130XL or 3500XL) (Applied Biosystems, Foster City, CA, USA) using the coffalyser software (MRC Holland, Amsterdam, The Netherlands).

**Gene Panel sequencing**

For Gene Panel analysis, DNA samples were sequenced on a next-generation sequencing platform with a collaboration partner (Centogene AG, Rostock, Germany). 29 genes previously linked to PD or dystonia (DYT) phenotypes were analyzed. The mean sequencing depth was 600x. Variants were filtered according to quality scores (cut-offs: quality score >200; coverage >10, allele fraction >20%), further filtered for the number of times that they appeared in public databases (cut-off: <0.01 in public databases and the in-house database) and finally for protein-changing variants in PD genes. Candidate variants were confirmed by Sanger sequencing. All genes included in the gene panel are disclosed in **Table S2**.

**RNA isolation**

Total RNA was isolated from human midbrain samples using TRIzol (Invitrogen) following the manufacturer’s instructions. 1 ml of TRIzol was added to each midbrain sample, followed by 100 µL of 1-Bromo-3-Chlor-Propane (Sigma Aldrich). Samples were mixed by inversion and the lysates were centrifuged at 12.000 × g for 15 min at 4°C, for organic/aqueous phase separation. The RNA-containing aqueous phase was isolated and RNA precipitation was performed with 500 µl of 2-propanol (AppliChem) and 2 µl GlycoBlue Co-precipitant (15 mg/ml)(ThermoFisher). Next, the samples were centrifuged at 12.000 × g for 30 min at 4°C. RNA pellets were washed three times with 75% ice-cold ethanol (AppliChem). The pellets were dried for 5 min, and reconstituted with 15-20 µl of nuclease-free water (Sigma Aldrich). RNA samples were incubated at 55°C for 2 min in order to completely dissolve the RNA. Finally, DNAse treatment (Life Technologies) was performed in accordance with the manufacturer’s instructions, and the RNA samples were cleaned and concentrated with the RNA Clean & Concentrator-5 KIT (Zymo Research). RNA integrity was assessed with the Agilent 6000 Nano Kit in the 2100 Bioanalyzer (Agilent).

**Preparation of RNA Sequencing Libraries**

The same RNA source was used for both small and total RNA sequencing experiments. Small RNA libraries were prepared using the TruSeq SmallRNA Library Prep Kit (Illumina) with minor modifications: 600 ng total RNA were used as starting material for library preparation. In order to prevent the formation of adapter dimers (by 5΄ and 3΄ self-ligation) and consequent amplification of these dimers, the CleanTag Library Preparation for Next-Generation Sequencing Kit (TriLink, San Diego, CA, USA) was employed. Total RNA libraries were prepared using a modified version of the TruSeq Stranded Total RNA protocol (Illumina), a strand-specific, massive-parallel cDNA sequencing protocol. 200 ng of total RNA were used as a start material. A ribosomal RNA (rRNA) depletion protocol (RiboMinus™, ThermoFisher) was performed in order to maintain rRNA content under 5% in the samples. Next, an adaptor ligation step is performed, followed by PCR amplification of the reads. A reduced number of PCR cycles was employed in order to avoid PCR duplication artifacts, as well as primer dimers in the final libraries. The standard sensitivity RNA Analysis Kit was used for Fragment Analyzer runs. For accurate quantification of small RNA libraries, a library pool was quantified with the QuantiFluor dsDNA System (Promega). cDNA library sizes were determined with the dsDNA 905 Kit (Agilent).

**RNA sequencing raw data processing and mapping**

After small and total RNA sequencing, the raw data was processed with a customized in-house pipeline (1). Adapter trimming and demultiplexing were then performed alongside base calling. For the small RNA sequencing data, the 3' adapters were trimmed and reads with the minimum length of 16 nucleotides were filtered out with the Cutadapt pipeline (2). The reads were then mapped to the reference genome for miRNAs/piRNAs known sequences, followed by other small non-coding RNAs. The remaining unmapped reads were mapped to the human genome. Bowtie version 1.1.2 (3) was employed for all mapping steps, and no mismatches were allowed for reads ≤ 32 b. For reads between 33 b and 50 b, one mismatch was tolerated. For total RNA sequencing data, RNA reads were mapped to the human transcriptome using RNA-STAR version STAR_2.5.2b (4) for all mapping steps. The reads were mapped in the non-splice-junction-aware mode and no mismatches for the reads <19 b were allowed. For reads between 20b and 39b, one mismatch was allowed, and for reads between 40b and 59b, two mismatches were tolerated. Besides the parameters depicted here, all other parameters were set as default in RNA-STAR. Aligned reads overlapping exons (for each gene) was counted with the intersection-non-empty mode of the htseq-count script (HTSeq package version 0.9.1)(5).

**Preparation of peptide libraries, SWATH-MS Run Settings and data mapping**

For proteomics experiments, human midbrain tissue lysis was performed with Urea/Thiourea/Chaps lysis buffer. Samples were homogenized and sonicated for 30s intervals and amplitude of 40%. Protein quantification was performed with the modified-Bradford Roti-Nanoquant Kit (Carl-Roth). Sample electrophoresis was performed previously to mass spectrometric experiments as a cleanup step. For this, samples were run a short distance (ca. 1 cm) into SDS-PAGE, each sample (‘lane’) was cut out as a single piece, and contained proteins were digested with trypsin for subsequent analysis. For the generation of peptide libraries, equal aliquots from each sample were pooled to a total of 80 µg and further separated into eight fractions using a reversed-phase spin column (Pierce High pH Reversed-Phase Peptide Fractionation Kit) (ThermoFisher). Spike-ins from a synthetic peptide standard were added to the samples and used for retention time alignment (iRT Standard). Protein digests were analysed on the Eksigent nanoLC425 nanoﬂow chromatography system (AB Sciex), hyphenated to a hybrid triple quadrupole-TOF mass spectrometer (TripleTOF 5600+) equipped with a Nanospray III ion source (Ionspray Voltage 2400 V, Interface Heater Temperature 150°C, Sheath Gas Setting 12) and controlled by Analyst TF 1.7.1 software build 1163 (AB Sciex). Peptides were dissolved in a loading buffer (composed of 2% acetonitrile and 0.1% formic acid in water). Following column enrichment, separation was performed on an analytical RP-C18 column of dimensions 0.075 mm ID x 250 mm, HSS T3, 1.8 µm (Waters, Milford, MA, USA) using a 90 min linear gradient of 5-35 % acetonitrile / 0.1% formic acid (v:v) at 300 nl min-1. Qualitative liquid chromatography/tandem mass spectrometry (LC-MS/MS) analysis was performed using a Top25 data-dependent acquisition method. For that, an MS survey scan of m/z 350–1250 accumulated for 350 ms at a resolution of 30,000 full width at half maximum (FWHM) was performed. MS/MS scans of m/z 180–1600 were accumulated for 100 ms at a resolution of 17,500 FWHM and a precursor isolation width of 0.7 FWHM, resulting in a total cycle time of 2.9 s. Precursors above a threshold of 125 cps for MS intensity and with charge states of 2+ / 3+ / 4+ were selected for MS/MS. Dynamic exclusion time was set to 30 s. An MS/MS activation was achieved by CID using nitrogen as a collision gas. For quantitative sequential window acquisition of all theoretical fragment ion spectra (SWATH) analysis, MS/MS data were acquired using 65 variable size windows across the 400-1,050 m/z range. Fragments were produced using rolling collision energy settings for charge state 2+, and fragments acquired over an m/z range of 350–1400 for 40 ms per segment. With the inclusion of a 100 ms survey scan, the overall cycle time was of 2.75s. A total of 407,752 MS/MS spectra from the combined qualitative analyses were searched against the UniProtKB human reference proteome (revision 04/2018, 93.609 entries) augmented with a set of 52 known common laboratory contaminants.

**Reverse Transcription and Quantitative Real-Time Polymerase Chain Reaction**

Fresh RNA was isolated from each midbrain sample from the discovery cohort for the experimental validation of selected targets from small RNA and total RNA sequencing results (see RNA isolation method above). RNA samples were reverse transcribed using the Quantitect RT Kit (Qiagen) for total RNAs and the miRCURY LNA RT Kit (Qiagen) for miRNAs. Both techniques were performed using the same RNA source as starting material. Quantitative real time polymerase chain reaction (qRT-PCR) experiments were performed in 45 cycles on a QuantStudio5 system (Applied Biosystems), using either the Quantitect SYBR Green PCR Master Mix (Qiagen) or the miRCURY LNA SYBR Green PCR Master Mix (Qiagen). All primers were designed with the GeneGlobe Design and Analysis Hub platform from Qiagen (6) [[92]](https://www.zotero.org/google-docs/?T5V2ox). Abundance was normalized with expression values of applicable housekeeping genes (HKG) (i.e. GAPDH) and relative expression levels (RELs) were calculated using the ΔCt method (ΔCt = CtGeneOfIntterest – CtHKG; REL = 2-ΔCt).

**Protein Isolation, Quantification and Western Blotting**

For the validation of proteomics results, fresh proteins were isolated from each midbrain sample from the discovery cohort using RIPA lysis buffer (ThermoFisher) adding protease and phosphatase inhibitors (CPI 1:25 / PhosSTOP 1:20) (Roche). No postmortem material was available for one PD subject and one CTR subject for protein isolation. Therefore, the final cohort numbers for Western Blotting experiments accounted to 12 PD and 9 CTR subjects. After lysis, samples were homogenized several times with a fine needle (BD Microlance 3, 26G x ⅜”, Becton-Dickinson). Total protein concentrations were determined with the Pierce BCA assay (ThermoFisher). For that, 200 μl of BCA reagent were pipetted to 1 μl of each sample. 20μl of the protein standard (Pierce Bovine Serum Albumin, 2 mg/ml standard, ThermoFisher) were pipetted in triplicates to a flat bottom 96-well plate (Corning). Standard volumes were diluted subsequently in order to make a 7-point 1:2 standard curve. After incubation (30 min/37 ºC), the absorption of each well was measured in a spectrophotometer (Infinite M200 Pro, Tecan) at the wavelength of 562 nm. Protein concentrations were calculated based on standard values with the given absorbances. Gel electrophoresis and protein electroblotting was performed with the NuPAGE system (Invitrogen) following previously published protocols (7) with slight modifications. Briefly, sodium dodecyl sulfate-polyacrylamide gel electrophoresis (SDS-PAGE) was performed with NuPAGE Bis-Tris precast gels (Invitrogen) using Lithium dodecyl sulfate (LDS) sample loading buffer added of 20 mM DTT reducing agent (Invitrogen). 30 μg of protein were loaded in each well of the stacking gels. PageRuler Prestained Protein Ladder (Invitrogen) was loaded as a molecular weight marker for every gel. Proteins were separated by electrophoresis with the NuPAGE MES SDS Running Buffer in a NuPAGE Mini Gel tank system (200V/35 min). Next, proteins were transferred to a PVDF (Poly(vinylidene fluoride)) membrane using the iBlot 2 Dry Blotting System (Invitrogen) following the manufacturer’s instructions. For each protein of interest, all protein samples were loaded/processed at the same time in a pair of NuPAGE Bis-Tris gels run in the same electrophoretic tank and electroblotted to one single PVDF membrane. For immunodetection, PVDF membranes were blocked in 5% non-fat milk diluted in tris-buffered saline, added of 0.05% Tween 20 (TBS-T 0.05%). Primary antibodies were added in blocking buffer in specific dilutions (see below), and tubes were incubated overnight at 4°C under agitation. According to the primary antibody used, either anti-rabbit or anti-mouse HRP-conjugated secondary antibodies were applied at 1:10.000 dilution (in blocking buffer) and incubated for 1 hour at room temperature under agitation. For western blot band detection, SuperSignal West Pico PLUS Chemiluminescent Substrate (ThermoFisher) was used, and the membranes were imaged in an Fusion SL chemiluminescence imaging system (Vilber Lourmat). Finally, quantification of the intensity of the bands was done using ImageJ. Signal intensities for the proteins of interest were normalized to the bands of the respective housekeeping proteins, as well as to the average intensity of all bands for the housekeeping protein across the pair of gels processed together. Primary antibodies included: HSPA1B Polyclonal Antibody (PA597846, 1:1.000, Invitrogen); [USP12 Polyclonal Antibody](https://www.thermofisher.com/order/catalog/product/PA568439) (PA568439, 1:1.000, Invitrogen); [ALDH1A1 Polyclonal Antibody](https://www.thermofisher.com/order/catalog/product/PA534623) (PA534623, 1:3.000 Invitrogen); [FNIP2 Polyclonal Antibody](https://www.thermofisher.com/order/catalog/product/PA520690) (PA520690, 1:500, Invitrogen); [CHI3L1 Polyclonal Antibod](https://www.thermofisher.com/order/catalog/product/PA543746)y (PA543746, 1:500, Invitrogen), Tyrosine Hydroxylase Polyclonal Antibody (PA585167, 1:1.000 Invitrogen); GAPDH Loading Control Monoclonal Antibody (MA515738, 1:2.000, Invitrogen); ß-actin Monoclonal Antibody (#4970, 1:4.000 Cell Signalling). Secondary antibodies included: Goat Anti-Rabbit IgG H&L secondary antibody (Ab205718, 1:10.000, Abcam) and Horse Anti-Mouse IgG H&L secondary antibody (PI-2000-1; 1:10:000, VectorLabs).

**Unsupervised visualizations**

The top 100 most variant omics were identified using the rowVars function available in the matrixStats package in R software (8), followed by a z-score transformation. Heatmaps were constructed by a complete hierarchical clustering with Euclidean distance over all samples, and illustrated using the ComplexHeatmap visualization package (9). Furthermore, pairwise sample correlation analyses for small and total RNA sequencing, and proteomics expression were performed using a multinomial Bayesian Hierarchical Clustering via the bhc function with default parameters (10). For this, the PD samples of the discovery cohort and normalized mapped counts without any additional transformation were used as input (10,11). Furthermore, high-dimensional reduction techniques Principal Component Analysis (PCA) and t-SNE (t-distributed stochastic neighbor embedding) were employed using the plotPCA (12) and Rtsne (13) functions on the RNA sequencing data, respectively.

**RNA library quality and statistical correlation with clinical parameters**

Clinical parameters including RNA integrity (RIN), Post-mortem interval (PMI) values, sex and disease duration were collected from all cohort samples. Similarly, quality control metrics like per sequence GC content (guanine-cytosine content) and number of total sequences, were retrieved from each FASTQ raw sequencing file as reported by FastQC tools (14). To establish the effect of these clinical parameters on technical library quality, correlation and statistical inferences between variables were represented by Pearson Correlation coefficient (R) and Student’s t-test, computed via the ggpubr R package (15) functions cor and cor.test, respectively. Further, linear relationships between these clinical parameters and disease duration of PD samples, against validated proteins, were predicted using the lm function from stats R package (8), and quantified by squared Pearson Correlation (R2) and Student’s t-test. Values were considered significant by p-value < 0.05.

**Pre-processing of small and total RNA sequencing data**

Two distinct pre-processing procedures to remove low-count and low-variance data were considered. Framework A starts with an initial elimination of all zero-count-row entrances of the count matrix, and is followed by the removal of low-count small RNAs/genes based on their data distribution using a quantile filtering with a cutoff of 25% and 50%, respectively, for the small RNA-seq and total RNA-seq data, using the TCGAanalyze Filtering function available in the R package TCGAbiolinks (16). In contrast, framework B initializes by estimating the unwanted variance in the given matrix count of genes/small RNA-seq and proceeded with the identification and removal of house-keeping-genes, i.e. a set of genes whose expression is not influenced by the conditions of the study, using the Remove Unwanted Variance (RUV) available in the R package RUVSeq (17). Further, the raw counts are transformed into log-form counts by assuming a Negative-Binomial (NB) distribution using the Variance Stabilizing Transformation (VST) (18) method for unsupervised data visualization.

**Identification of sex-specific genes and differential expression results**

In order to control for sex-related effects in the differential expression results, we modelled transcriptomics data taking into consideration sex as a covariate. To identify sex-specific genes, genes from the Y chromosome were retrieved from the ENSEMBL database (19) (genome build CRCh38.p13), and additional sex-differential expression comparison between sex conditions was established. Both analyses were performed using DESeq2 (20) under significance criteria of adjusted p-value < 0.1.

**RNA-seq decomposition**

In an attempt to make an assessment on the proportion of neuronal and immune cells present in the different cohorts (PD patients and control), we leveraged the RNASeq Decomposition computational tools SCADEN (Single-cell Assisted Deconvolutional Network) (21) and the method presented by Finotello and Trajanoski (22). The former is a deep neural network framework trained on mouse brain single-cell RNA sequencing data (23) [.](https://www.zotero.org/google-docs/?tKShd3) To leverage this tool, we initially converted our HUGO Gene Nomenclature Committee (HGNC) identifiers to MGI (Mouse Genome Informatics) symbols through the getLDS function from the biomaRt R package (24, 25) (human and mouse versions GRCh38.p13 and GRCm39, respectively). The output proportion cell values (between 0 and 1) for each sample were reshaped to percentage values and normalised to equal 100% for each sample over 8 different neuronal cell classes (Astrocytes, Neurons, Oligodendrocytes, Oligodendrocyte Progenitor, Ependymal and Tanycytes, Vascular and Leptomeningeal, and Unknown). Significance between biological conditions for each cell class was tested using Student’s t-test. For the latter, we computed the TPM-transformed values of our original RNA-seq count data considering a gene length of 50 bp (see library preparation section) and leveraging the R function counts_to_tpm developed by Kamil Slowikowski (26). Considering we are interested in the comparison between cohorts, we applied the deconvolution methods *xcell* and *mcp-counter* since these are based on marker genes and therefore suitable for applications on data with different conditions. Further, mean values of the scores were calculated for each cell, and subsequent log_2_FoldChange (log_2_FC) between PD and control patients scores. Significance was introduced by a Wilcoxon t-test and succeeding computed adjusted p-values based on Bonferroni’s correction method. Significant cell scores were identified by adjusted p-value < 0.1.

**MiRNA target prediction and multi-omics data integration**

To predict miRNAs target genes, we used miRDB v22 (27) and mirTarBase 8.0 (28). The first consists in the application of support vector machines in high-throughput sequencing experiments, and the identification of new miRNA target binding and expression downregulation. The latter is based on text mining of functional studies of miRNAs and collects miRNA-target interactions (MTI) which have been experimentally validated. For miRDB, the default search criteria of target prediction score higher than 60 and miRNAs with less than 800 targets was considered to find all gene targets of all the miRNAs available in our data. In addition, integration between gene and protein expression was performed using the UniProt database (29). Furthermore, interacting pairs involving genes and proteins were expanded considering mutual direction and significance in either of these omics.

**WGCNA gene module analysis**

A weighted gene co-expression network analysis (WGCNA)(30) was performed to identify modules of interest, hub genes and explored modules’ pathways related to PD. The pre-processed normalised count data from DESeq2 was used to compute gene co-expression similarities from correlation measures and subsequently build an adjacency matrix. Thereafter, the matrix was converted to a weighted adjacency matrix by a power function of the correlation between genes. The power parameter ranging between 1 and 20 was investigated using the pickSoftThreshold function and defined based on a scale-free topology criterion higher than 0.8 (31). Subsequently, gene modules were detected by the blockwiseModules function with default parameters, and summarised by eigenvectors of the first principal component of the modules’ expression. For each module, module-trait (PD or control condition) relationships were estimated using a linear fit model and multiple test correction by functions lmFit and topTable with default parameters from Limma package (32), respectively. The modules of interest were identified by p-value < 0.05 and, subsequently, the genes in these key modules were integrated with validated miRNA targets (27) and visualised using Cytoscape (33).

**Gene ontology and pathway enrichment analyses**

To perform a GO term and pathway enrichment analyses of the differential expressed genes and genes in the WGCNA modules, we used ShinyGO (20) and several common functional annotation databases (i.e., GO and KEGG). This enrichment analysis was done separately for the differentially up- and down-regulated entities, and using a significance level of FDR < 0.1. Furthermore, protein-protein interaction (PPI) networks were generated and visualized with the STRING platform v.11 (21) using default settings.

**Statistical Analysis for experimentally validated molecular candidates**

Statistical analysis for the validation results of selected targets (qRT-PCR and Western Blotting results) was performed using GraphPad Prism 9. For both experimental approaches, data were shown as mean ± SEM. Normality tests were performed for the validation results of every selected target (D’agostino and Pearson and Shapiro-Wilk tests), and differences between PD and CTR cohorts were analyzed by either two-sided unpaired t-tests (in case data passed the normally tests) or Mann-Whitney-U tests (in case data did not pass the normality tests). For outlier detection, the ‘Robust regression and Outlier removal’ (ROUT) test (34) was applied (Q at 1%).

**SI References**

1. G. Jain, GJSrMap: The smallRNA mapping pipeline (GitHub, 2018).

2. M. Martin, Cutadapt removes adapter sequences from high-throughput sequencing reads. EMBnet j. 17, 10 (2011).

3. B. Langmead, C. Trapnell, M. Pop, S. L. Salzberg, Ultrafast and memory-efficient alignment of short DNA sequences to the human genome. Genome Biol 10, R25 (2009).

4. A. Dobin, et al., STAR: ultrafast universal RNA-seq aligner. Bioinformatics 29, 15–21 (2013).

5. S. Anders, P. T. Pyl, W. Huber, HTSeq--a Python framework to work with high-throughput sequencing data. Bioinformatics 31, 166–169 (2015).

6. GeneGlobe Design and Analysis Hub - Qiagen. Qiagen

7. Penna A, Cahalan M (2007) Western Blotting Using the Invitrogen NuPage Novex Bis Tris MiniGels. JoVE (Journal of Visualized Experiments) e264. https://doi.org/10.3791/264

8. R Core Team, R: A Language and Environment for Statistical Computing (R Foundation for Statistical Computing, 2017).

9 Z. Gu, R. Eils, M. Schlesner, Complex heatmaps reveal patterns and correlations in multidimensional genomic data. Bioinformatics 32, 2847–2849 (2016).

10. R. Savage, E. Cook, R. Darkins, Y. Xu, BHC: Bayesian Hierarchical Clustering (2019).

11. K. A. Heller, Z. Ghahramani, Bayesian hierarchical clustering in Proceedings of the 22nd International Conference on Machine Learning - ICML ’05, (ACM Press, 2005), pp. 297–304.

12. Huber W plotPCA: Sample PCA plot for transformed data in DESeq2: Differential gene expression analysis based on the negative binomial distribution

13. Van der Maaten L, Hinton G (2008) Visualizing data using t-SNE. Journal of machine learning research 9:

14. Andrews S, Krueger F, Segonds-Pichon A, et al (2010) FastQC. A quality control tool for high throughput sequence data. Babraham, UK

15. Kassambara A (2018) ggpubr:“ggplot2” based publication ready plots. R package version 01 7:

16. A. Colaprico, et al., TCGAbiolinks: an R/Bioconductor package for integrative analysis of TCGA data. Nucleic Acids Research 44, e71–e71 (2016).

17. D. Risso, J. Ngai, T. P. Speed, S. Dudoit, Normalization of RNA-seq data using factor analysis of control genes or samples. Nat Biotechnol 32, 896–902 (2014).

18. S. Anders, W. Huber, Differential expression analysis for sequence count data. Genome Biol 11, R106 (2010).

19. Howe, Kevin L., et al. "Ensembl 2021." Nucleic acids research 49.D1:D884-D891. (2021).

20. Love MI, Huber W, Anders S (2014) Moderated estimation of fold change and dispersion for RNA-seq data with DESeq2. Genome Biol 15:550. https://doi.org/10.1186/s13059-014-0550-8

21. Menden K, Marouf M, Oller S, et al (2020) Deep learning–based cell composition analysis from tissue expression profiles. Science advances 6:eaba2619

22. F. Finotello, Z. Trajanoski, Quantifying tumor-infiltrating immune cells from transcriptomics data. Cancer Immunol Immunother 67, 1031–1040 (2018).

23. Kiselev V, Andrews T, Hemberg M Collection of public scRNA-Seq datasets used by the Hemberg Lab

24. Durinck S, Spellman PT, Birney E, Huber W (2009) Mapping identifiers for the integration of genomic datasets with the R/Bioconductor package biomaRt. Nature protocols 4:1184–1191

25. Durinck S, Moreau Y, Kasprzyk A, et al (2005) BioMart and Bioconductor: a powerful link between biological databases and microarray data analysis. Bioinformatics 21:3439–3440

26. K. Slowikowski, Convert read counts to transcripts per million (TPM). (2016).

27. Y. Chen, X. Wang, miRDB: an online database for prediction of functional microRNA targets. Nucleic Acids Research 48, D127–D131 (2020).

28. H.-Y. Huang, et al., miRTarBase 2020: updates to the experimentally validated microRNA–target interaction database. Nucleic Acids Research, gkz896 (2019).

29. The UniProt Consortium, UniProt: a worldwide hub of protein knowledge. Nucleic Acids Research 47, D506–D515 (2019).

30. Langfelder P, Horvath S (2008) WGCNA: an R package for weighted correlation network analysis. BMC bioinformatics 9:1–13

31. Zhang B, Horvath S (2005) A general framework for weighted gene co-expression network analysis. Statistical applications in genetics and molecular biology 4:

32. Ritchie ME, Phipson B, Wu D, et al (2015) limma powers differential expression analyses for RNA-sequencing and microarray studies. Nucleic Acids Research 43:e47–e47. https://doi.org/10.1093/nar/gkv007

33. Shannon P, Markiel A, Ozier O, et al (2003) Cytoscape: a software environment for integrated models of biomolecular interaction networks. Genome research 13:2498–2504

34. Motulsky HJ, Brown RE (2006) Detecting outliers when fitting data with nonlinear regression–a new method based on robust nonlinear regression and the false discovery rate. BMC bioinformatics 7:1–20

**Supplementary tables**

**Appendix Table S1. Genes/Exons assessed in MLPA experiments**

| **Gene/Exon** | **Chromosomal band** | **hg18 location** |
| --- | --- | --- |
| TNFRSF9-2 | 01p36.23 | 01-007,923428 |
| PARK7-1 | 01p36.23 | 01-007,944030 |
| PARK7-1 | 01p36.23 | 01-007,944328 |
| PARK7-2 | 01p36.23 | 01-007,945409 |
| PARK7-3 | 01p36.23 | 01-007,947986 |
| PARK7-4 | 01p36.23 | 01-007,951947 |
| PARK7-5 | 01p36.23 | 01-007,953554 |
| PARK7-6 | 01p36.23 | 01-007,960310 |
| PARK7-7 | 01p36.23 | 01-007,967679 |
| ATP13A2-9 | 01p36.13 | 01-017,199465 |
| ATP13A2-2 | 01p36.13 | 01-017,204821 |
| PINK1-1 | 01p36.12 | 01-020,832858 |
| PINK1-2 | 01p36.12 | 01-020,836975 |
| PINK1-3 | 01p36.12 | 01-020,839042 |
| PINK1-4 | 01p36.12 | 01-020,843555 |
| PINK1-5 | 01p36.12 | 01-020,844754 |
| PINK1-6 | 01p36.12 | 01-020,847671 |
| PINK1-7 | 01p36.12 | 01-020,848135 |
| PINK1-8 | 01p36.12 | 01-020,850116 |
| SNCA-6 | 04q22.1 | 04-090,866748 |
| SNCA-5 | 04q22.1 | 04-090,869382 |
| SNCA-4 | 04q22.1 | 04-090,962507 |
| SNCA-3 | 04q22.1 | 04-090,968331 |
| SNCA-2 (A30P) | 04q22.1 | 04-090,975721 |
| SNCA-2 | 04q22.1 | 04-090,975795 |
| SNCA-1 | 04q22.1 | 04-090,977117 |
| LPA-39 | 06q25.3 | 06-160,873567 |
| PARK2-12 | 06q26 | 06-161,690736 |
| PARK2-11 | 06q26 | 06-161,701126 |
| PARK2-10 | 06q26 | 06-161,727843 |
| PARK2-9 | 06q26 | 06-161,889882 |
| PARK2-8 | 06q26 | 06-161,910375 |
| PARK2-7 | 06q26 | 06-162,126840 |
| PARK2-6 | 06q26 | 06-162,314327 |
| PARK2-5 | 06q26 | 06-162,395114 |
| PARK2-4 | 06q26 | 06-162,542097 |
| PARK2-3 | 06q26 | 06-162,603527 |
| PARK2-2 | 06q26 | 06-162,784395 |
| PARK2-1 | 06q26 | 06-163,068646 |
| LRRK2-41 (G2019S) | 12q12 | 12-039,020438 |
| Reference | 02p13.2 | 02-071,750350 |
| Reference | 02q36.3 | 02-227,881969 |
| Reference | 03p22.2 | 03-038,494904 |
| Reference | 05q31.1 | 05-132,037610 |
| Reference | 06p12.3 | 06-051,605391 |
| Reference | 08q12.2 | 08-061,883322 |
| Reference | 09p13.3 | 09-034,483216 |
| Reference | 11p14.3 | 11-022,251032 |
| Reference | 15q21.1 | 15-042,674827 |
| Reference | 18q21.1 | 18-045,743292 |

**Appendix Table S2. List of genes assessed in Gene panel sequencing experiments**

| Targeted Sequencing (Gene Panel) - Evaluated Genes |
| --- |
| *ADCY5* |
| *ANO3* |
| *COX20* |
| *DJ-1* |
| *GBA* |
| *GCH1* |
| *GNAL* |
| *KMT2B* |
| *LRRK2* |
| *MCOLN1* |
| *Parkin* |
| *PDGFB* |
| *PDGFRB* |
| *PINK1* |
| *PLA2G6* |
| *POLG* |
| *PRKRA* |
| *RAB12* |
| *RAB39B* |
| *SGCE* |
| *SLC20A2* |
| *SNCA* |
| *TAF1 (4 variants)* |
| *THAP1* |
| *TOR1A* |
| *VAC14* |
| *VPS13C* |
| *VPS35* |
| *XPR1* |

**Appendix Table S3. RNA-Seq decomposition score results using xCell and MCP-counter methods.** Log_2_(Fold-Change) (Log_2_FC) = score_PD_ - score_CTR_, p-values obtained using a Wilcoxon t-test and adjusted by Bonferroni's correction method.

| **xCell** | | | |
| --- | --- | --- | --- |
| **Cell Type** | **Log_2_FC** | **p-value** | **p-adjusted value** |
| **Myeloid dendritic cell activated** | -2.185 | 9.483e-02 | 3.082e-01 |
| **T cell CD4+ (non-regulatory)** | 1.499 | 7.950e-02 | 2.819e-01 |
| **Granulocyte-monocyte progenitor** | 51.581 | 6.194e-02 | 2.416e-01 |
| **Class-switched memory B cell** | -1.895 | 5.863e-02 | 2.416e-01 |
| **T cell CD4+ central memory** | -2.355 | 5.393e-02 | 2.416e-01 |
| **B cell naive** | 3.711 | 4.430e-02 | 2.416e-01 |
| **T cell regulatory (Tregs)** | 3.359 | 2.968e-02 | 1.929e-01 |
| **Endothelial cell** | 2.607 | 2.696e-02 | 1.929e-01 |
| **B cell memory** | 2.512 | 2.324e-02 | 1.929e-01 |
| **Common myeloid progenitor** | -49.819 | 2.308e-02 | 1.929e-01 |
| **T cell CD4+ memory** | 4.524 | 1.429e-02 | 1.929e-01 |
| **Hematopoietic stem cell** | 3.566 | 7.761e-06 | 3.027e-04 |
| **MCP-counter** | | | |
| **Cell Type** | **Log_2_FC** | **p-value** | **p-adjusted value** |
| **T cell** | -6.025e-01 | 9.424e-02 | 5.183e-01 |
| **cytotoxicity score** | 1.021 | 8.350e-02 | 5.183e-01 |

**Appendix Table S4.** Effect of RNA integrity number (RIN) and post-mortem interval (PMI) on RNA library quality. Correlation of RIN and PMI values of the whole cohort and % of GC content and number of total sequences. Pearson correlation coefficient (R) and p-values from Student’s t-test are displayed.

|  | **% GC content** | **Number of total sequences** |
| --- | --- | --- |
| **RIN** | R = 0.118  (p-value = 0.591) | R = 0.099  (p-value = 0.653) |
| **PMI** | R = -0.135  (p-value = 0.538) | R = 0.221  (p-value = 0.311) |

**Appendix Table S5.** Effect of RNA integrity number (RIN) and post-mortem interval (PMI) on deregulated proteins of interest. Linear regression of RIN, PMI and disease duration (in years) values in PD patients against normalized proteomics counts of significant proteins from validation results Fig.S9. Square Pearson correlation (R2) and p-values from Student’s t-test are displayed.

|  | **CHI3L1** | **FNIP2** | **HSPA1B** | **TH** |
| --- | --- | --- | --- | --- |
| **RIN** | R2 = 8.28 x 10-3  (p-value = 0.767) | R2 = 2.65 x 10-2  (p-value = 0.595) | R2 = 3.84 x 10-2  (p-value = 0.521) | R2 = 2.41 x 10-1  (p-value = 0.088) |
| **PMI** | R2 = 8.61 x 10-3  (p-value = 0.763) | R2 = 9.86 x 10-2  (p-value = 0.296) | R2 = 1.49 x 10-1  (p-value = 0.193) | R2 = 1.14 x 10-4  (p-value = 0.972) |
| **Disease Duration** | R2 = 1.81 x 10-2  (p-value = 0.661) | R2 = 1.30 x 10-3  (p-value = 0.907) | R2 = 5.14 x 10-3  (p-value = 0.816) | R2 = 1.41 x 10-1  (p-value = 0.206) |

**Appendix Table S6. Integration pairs between significantly DE miRNAs and predicted/validated mRNA/gene targets from framework B.**

| **Validated targets - mirTarBase** | | | | | | | |
| --- | --- | --- | --- | --- | --- | --- | --- |
| **Gene Name** | **miRNA** | **log_2_FC miRNA** | **p-value miRNA** | **padj miRNA** | **log_2_FC gene** | **p-value gene** | **padj gene** |
| CREBBP | hsa-miR-218-5p | 6.366e-01 | 2.394e-04 | 9.122e-02 | 3.513e-01 | 3.161e-04 | 9.181e-02 |
| PAPD7 | hsa-miR-218-5p | 6.366e-01 | 2.394e-04 | 9.122e-02 | 3.802e-01 | 5.215e-04 | 9.934e-02 |
| POU2F2 | hsa-miR-218-5p | 6.366e-01 | 2.394e-04 | 9.122e-02 | 8.656e-01 | 3.785e-04 | 9.181e-02 |
| RBM6 | hsa-miR-218-5p | 6.366e-01 | 2.394e-04 | 9.122e-02 | 4.343e-01 | 4.046e-04 | 9.181e-02 |
| **Predicted targets - miRDB** | | | | | | | |
| **Gene Name** | **miRNA** | **log_2_FC miRNA** | **p-value miRNA** | **padj miRNA** | **log_2_FC gene** | **p-value gene** | **padj gene** |
| ETV5 | hsa-miR-369-3p | 6.984e-01 | 1.862e-04 | 9.122e-02 | 7.051e-01 | 5.476e-04 | 9.934e-02 |
| GTF2H3 | hsa-miR-369-3p | 6.984e-01 | 1.862e-04 | 9.122e-02 | -4.633e-01 | 2.225e-04 | 4.081e-02 |
| JADE2 | hsa-miR-218-5p | 6.366e-01 | 2.394e-04 | 9.122e-02 | 6.022e-01 | 4.012e-04 | 9.181e-02 |
| RAB6C | hsa-miR-218-5p | 6.366e-01 | 2.394e-04 | 9.122e-02 | -8.860e-01 | 3.331e-04 | 9.181e-02 |
| RCOR1 | hsa-miR-218-5p | 6.366e-01 | 2.394e-04 | 9.122e-02 | 3.516e-01 | 6.048e-04 | 9.933e-02 |
| SLC38A2 | hsa-miR-369-3p | 6.984e-01 | 1.862e-04 | 9.122e-02 | 9.138e-01 | 1.561e-04 | 7.404e-02 |

**Appendix Table S7.** Overview of differential expression and integration results of all omics with different log_2_FC thresholds for frameworks A and B.

| **Differential Expressed (DE) Omics (p-adjusted < 0.1)** | | | | | |
| --- | --- | --- | --- | --- | --- |
| **log_2_FC thresholds** | **Genes - framework A** | **Genes - framework B** | **miRNAs - framework A** | **miRNAs - framework B** | **Proteins** |
| **log_2_FC = 0** | 641 | 126 | 0 | 4 | 22 |
| **log_2_FC = 0.3** | 627 | 126 | 0 | 4 | 21 |
| **log_2_FC = 0.5** | 543 | 114 | 0 | 4 | 18 |

| **Pairs of DE genes and miRNAs targets** | | | | |
| --- | --- | --- | --- | --- |
| **log_2_FC thresholds** | **Down-regulated miRNA - framework A** | **Up-regulated miRNA - framework A** | **Down-regulated miRNA - framework B** | **Up-regulated miRNA - framework A** |
| **log_2_FC = 0** | 2,250 | 2,545 | 336 | 464 |
| **log_2_FC = 0.3** | 771 | 970 | 43 | 89 |
| **log_2_FC = 0.5** | 281 | 484 | 7 | 14 |

| **Pairs of genes, proteins and miRNAs targets** | | | | |
| --- | --- | --- | --- | --- |
| **log_2_FC thresholds** | **Down-regulated miRNA - framework A** | **Up-regulated miRNA - framework A** | **Down-regulated miRNA - framework B** | **Up-regulated miRNA - framework A** |
| **log_2_FC = 0** | 12,943 | 10,164 | 11,556 | 11,992 |
| **log_2_FC = 0.3** | 283 | 260 | 58 | 74 |
| **log_2_FC = 0.5** | 12 | 17 | 3 | 2 |

**Supplementary Figures**


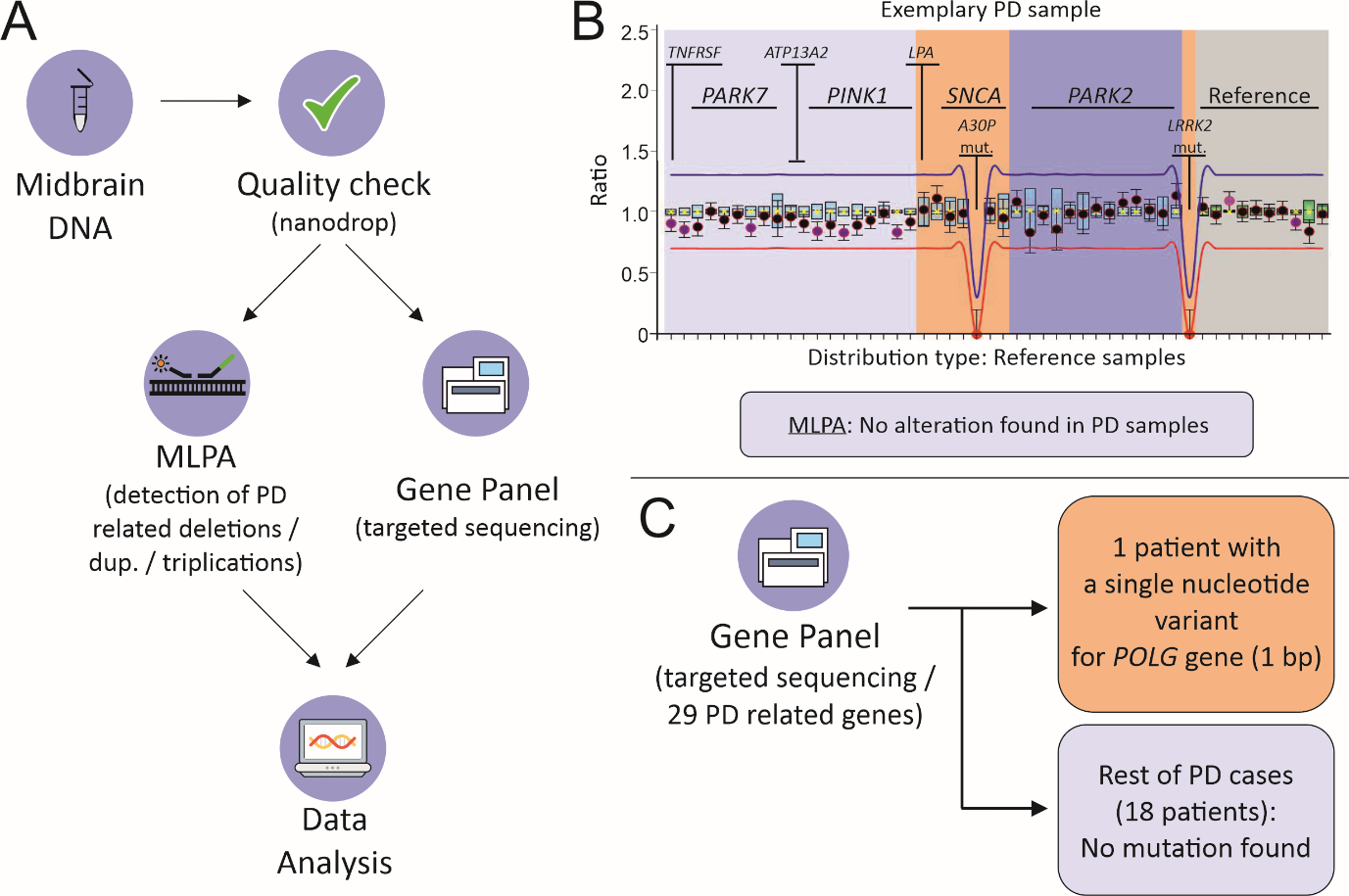


**Appendix Figure S1. Gene panel and MLPA experiments overview. (A)** Experimental design. After quality check, DNA samples were processed in MLPA / Gene panel sequencing experiments. **(B)** Exemplary MLPA results for a PD patient showing no alteration (deletion, duplications or triplications) for the explored genes. Likewise, no alterations were found in the PD patient cohort. **(C)** Gene panel experiments reveal a single nucleotide variant (length: 1 bp) for the *POLG* gene in one of the PD patients. No mutations found for the remaining cohort.


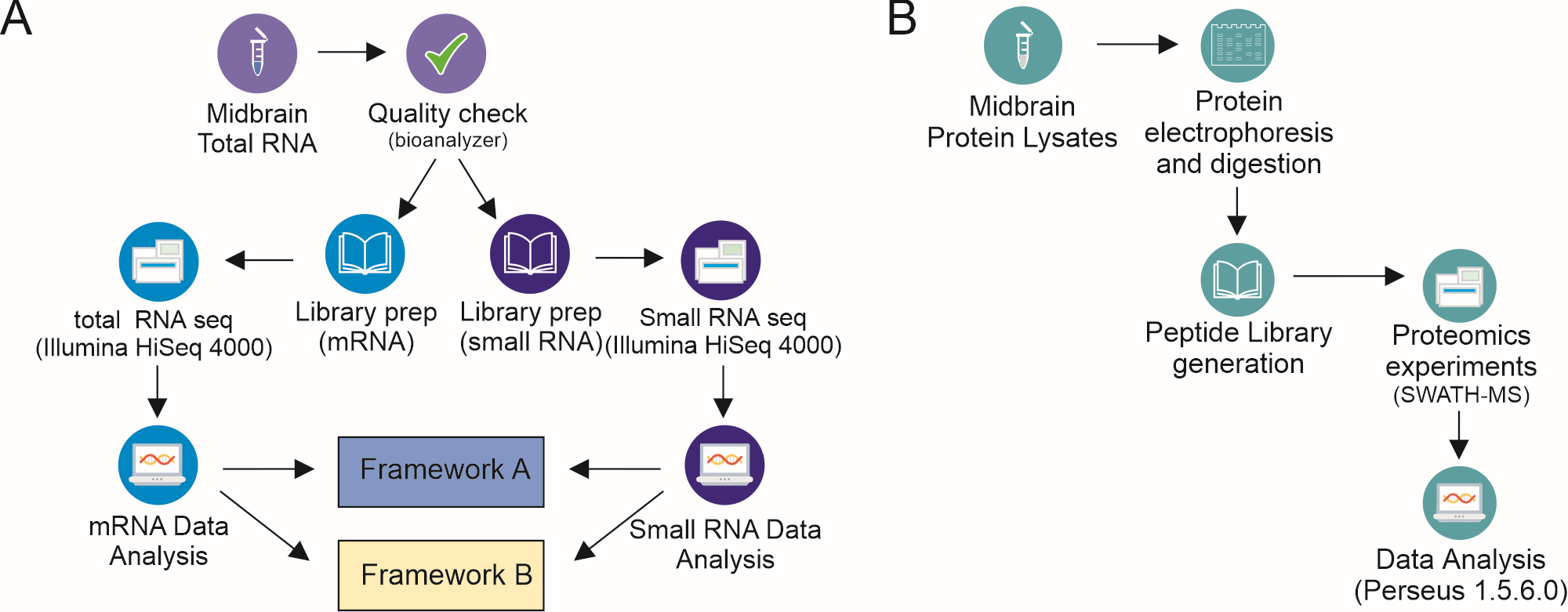


**Appendix Figure S2. Experimental design for RNA sequencing and proteomics experiments. (A)** After quality check, small and total RNA libraries were prepared from each midbrain sample. Sequencing was performed in the Illumina HiSeq 400 platform, followed by bioinformatic analysis with two different frameworks (“A” and “B”). **(B)** Proteins extracted from midbrain tissue were separated by electrophoresis and digested by trypsinization. Peptide libraries were generated and Sequential Window Acquisition of all Theoretical Mass Spectra (SWATH-MS) experiments were performed, followed by data analysis using Perseus 1.5.6.0.

**
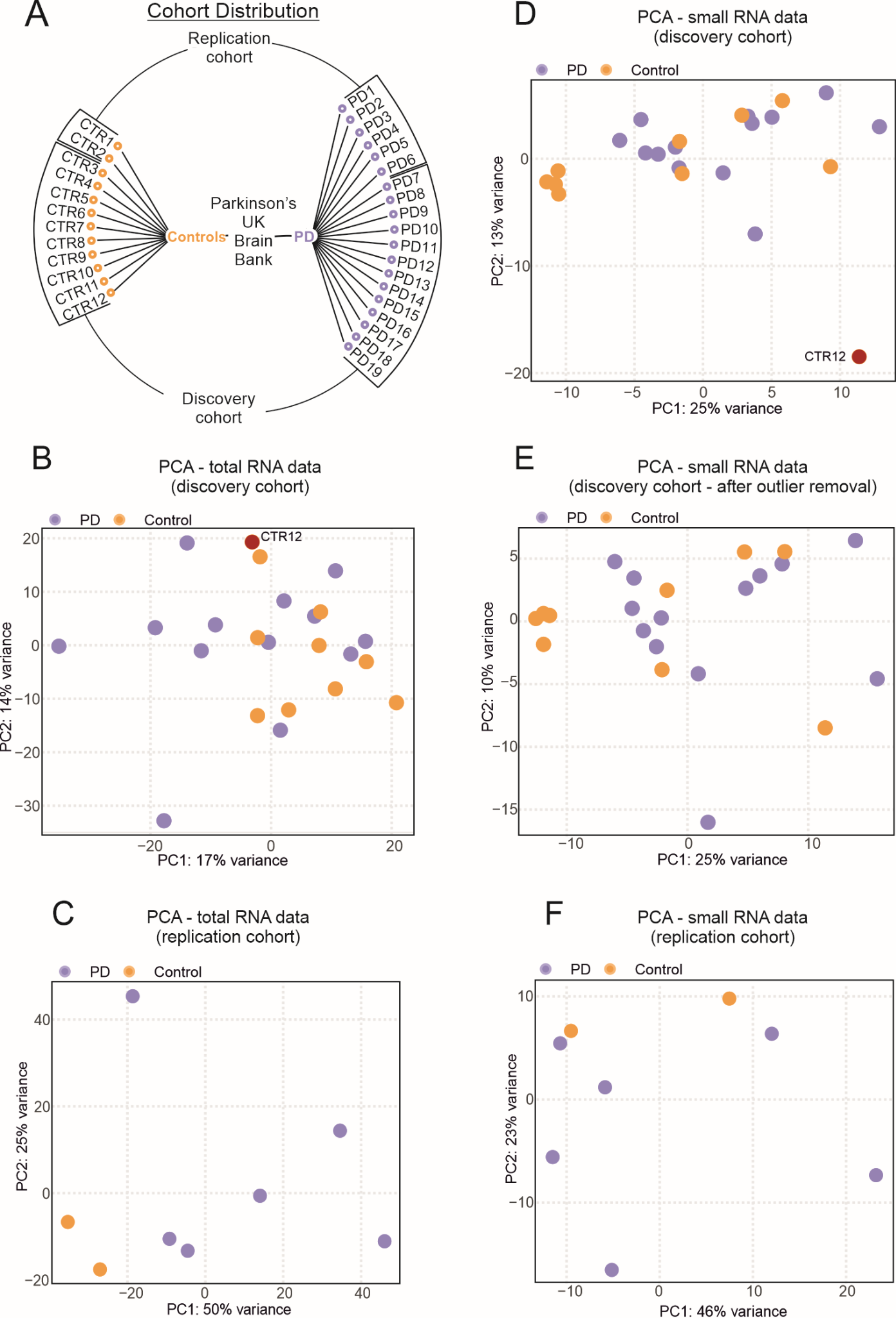
**

**Appendix Figure S3. Cohort composition and Principal Component Analyses (PCA) for RNA Sequencing experiments. (A)** Cohort composition for the human postmortem midbrain samples obtained from the Parkinson’s UK Brain Bank. The outlines indicate the samples from the discovery cohort, composed by 13 Parkinson’s disease (PD) patients and 10 controls (CTR), and the replication cohort, which included 6 PD and 2 CTR. **(B)** PCA for the total RNA data for the discovery cohort, with the control sample CTR12 removed from the analysis for the small RNA-seq data coloured in red. **(C)** PCA for the total RNA data for the replication cohort. **(D)** PCA for the small RNA data for the discovery cohort. Outlier sample CTR12 lies separately from the rest of the samples (details in the Results section). **(E)** PCA for small RNA data for the discovery cohort after outlier removal. **(F)** PCA for the small RNA data for the replication cohort.**
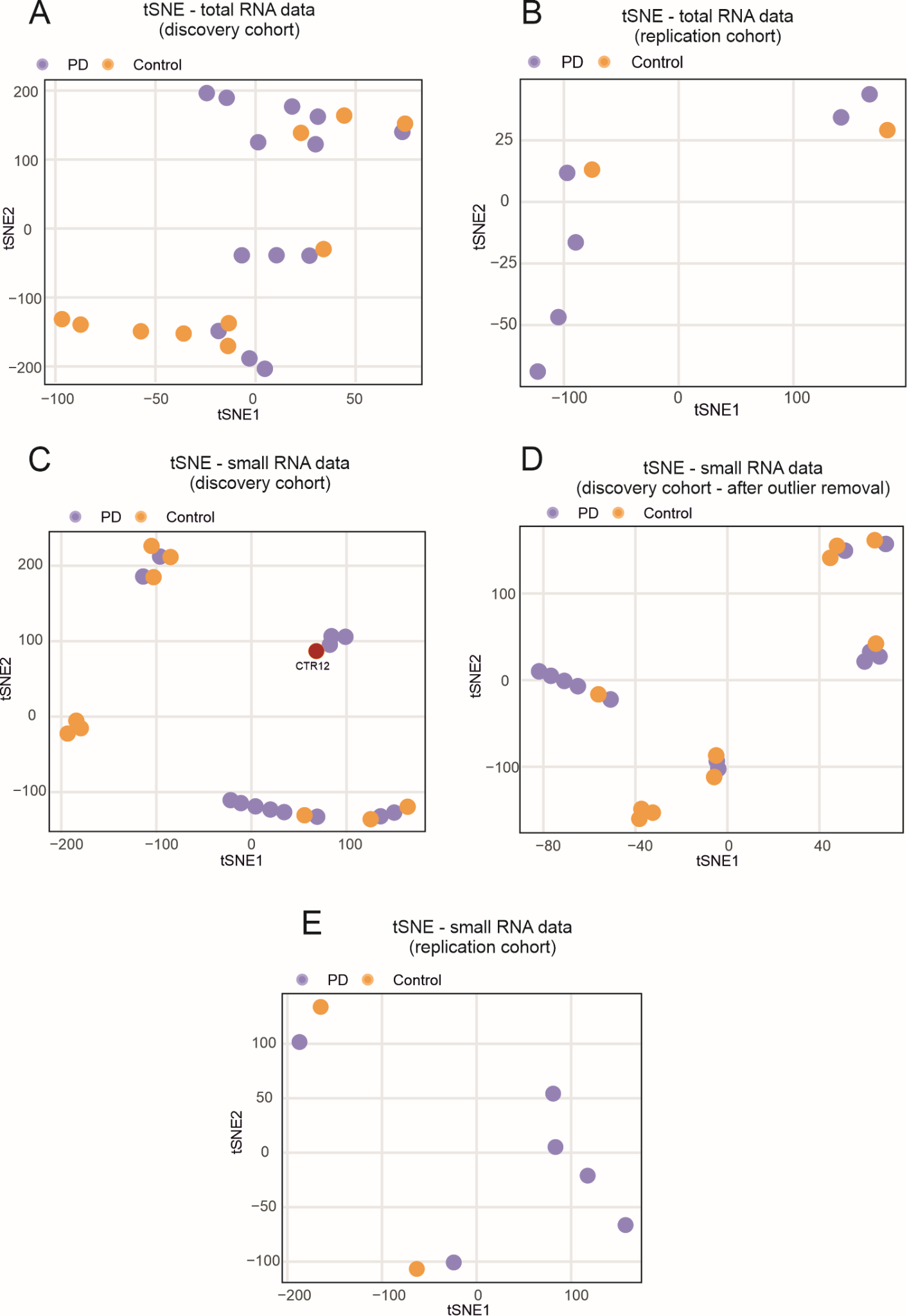
**

**Appendix Figure S4. T-SNE representations of (A)-(B) total RNA and (C)-(E) small RNA sequencing data, for discovery and replication cohorts.** Sequencing data contains 13 PD and 10 CTR, and 6 PD and 2 CTR patients within the discovery and replication cohorts, respectively. In **(C)** is highlighted the outlier control sample CTR12 in the smallRNA sequencing data, and in **(D)** the resulting representation followed by outlier removal.

**
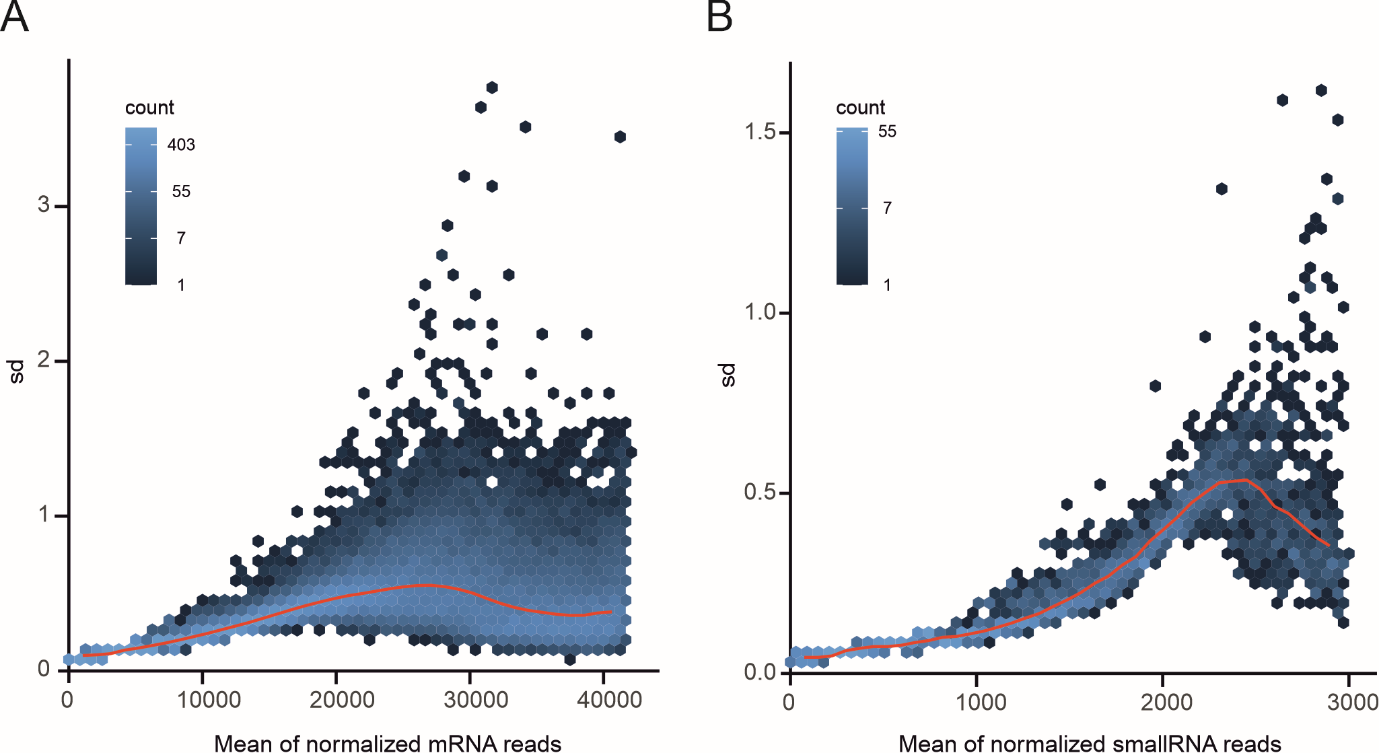
**

**Appendix Figure S5. Mean dispersion estimation plots for (A) mRNA and (B) smallRNA count reads.** The y-axis represents the standard deviation (sd) of the mean counts distribution, the color scale indicates the mean count intensity, and the red curve is the fit between dispersion and average through maximum likelihood estimates (MLEs).


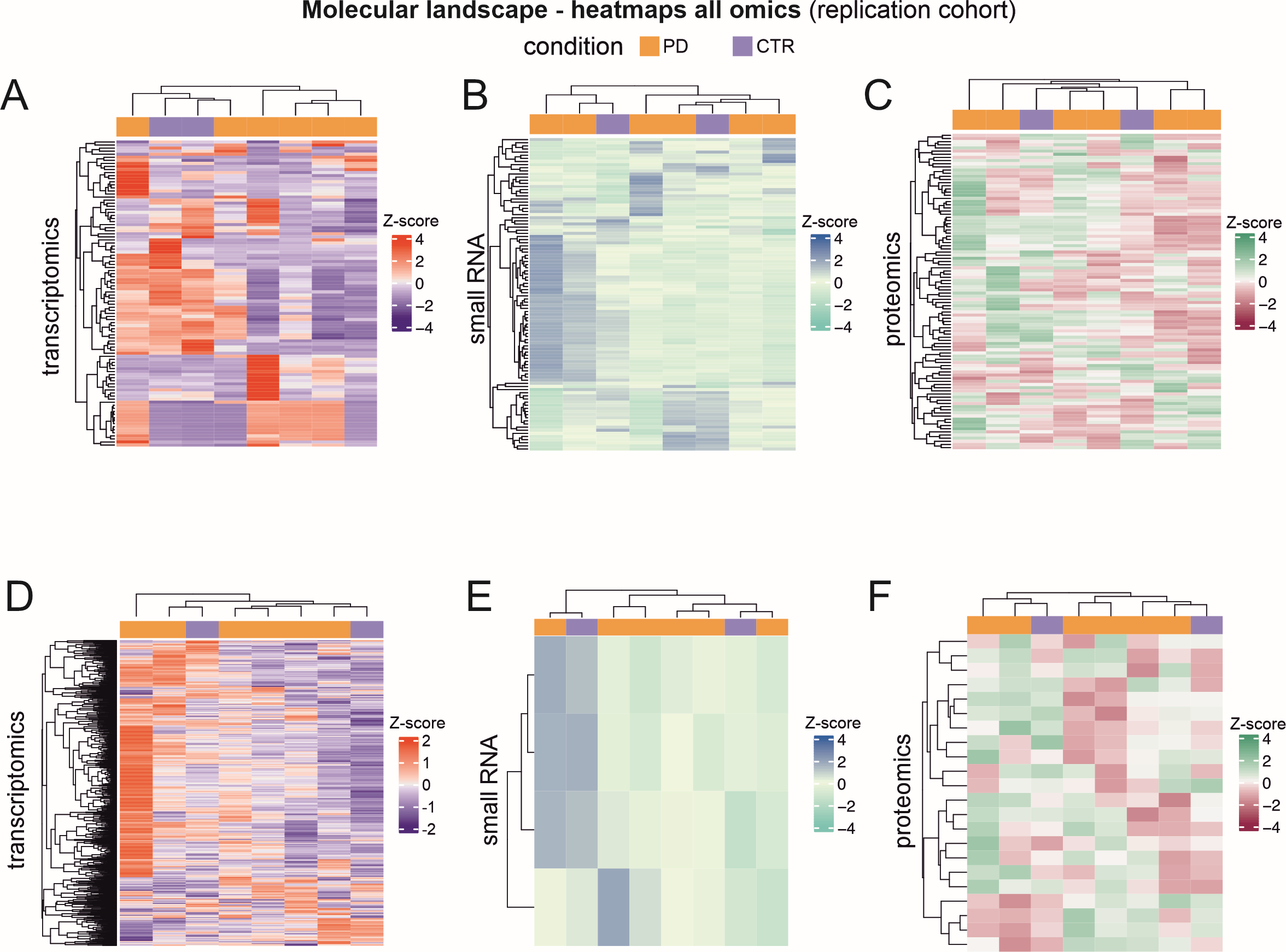


Appendix **Figure S6. Heatmaps of the 100 most variant and differentially expressed transcripts, small RNAs and proteins for frameworks A and B, for the replication cohort.** Heatmaps of the top 100 most variant transcripts, small RNAs and proteins are illustrated in **(A)-(C)**, respectively, for samples in the replication cohort. Based on frameworks A and B, a total of 667 genes, 4 miRNAs and 22 proteins were defined as differentially expressed (p-adjusted < 0.1). Their z-score normalized counts are displayed in **(D)-(F)**, respectively, for samples in the replication cohort. Each row represents an omic, and each column a PD or CTR sample. Cluster dendrograms were depicted from a complete Hierarchical with Euclidean distance clustering.

**
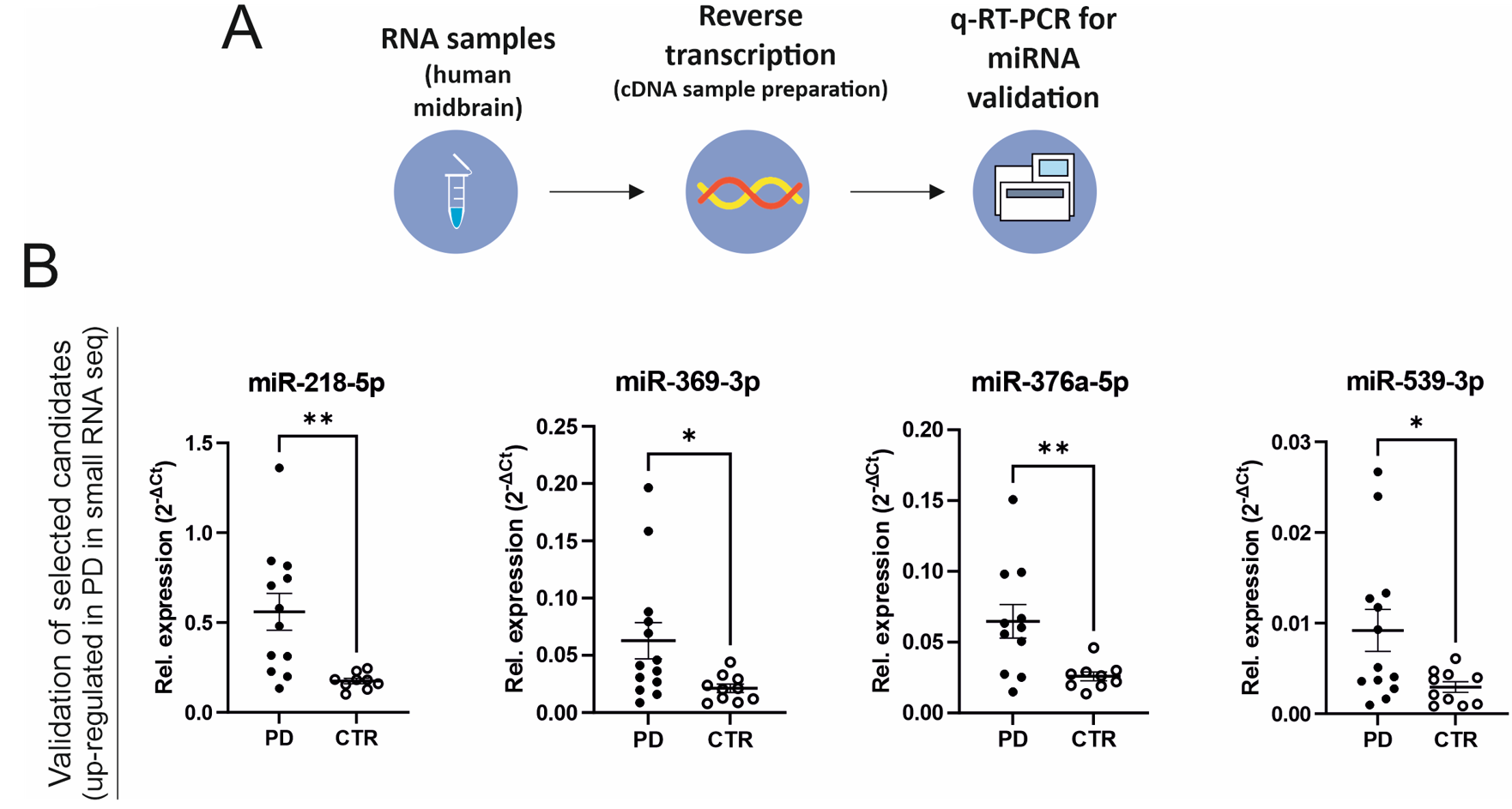
**

**Appendix Figure S7. Experimental setup and validation of small RNA sequencing results by qRT-PCR. (A)** Experimental setup for the validation of miRNA candidates. Reverse transcription was done with the miRCURY LNA RT Kit (Qiagen) and qRT-PCRs were conducted in the QuantStudio 5 platform. **(B)** Relative expression levels of miRNAs found up-regulated in PD patients by small RNA sequencing. Error bars show standard error of the mean. Data analyzed either by Mann-Whitney-U test or unpaired Student’s t-tests. * p < 0.05, ** p < 0.01.

**
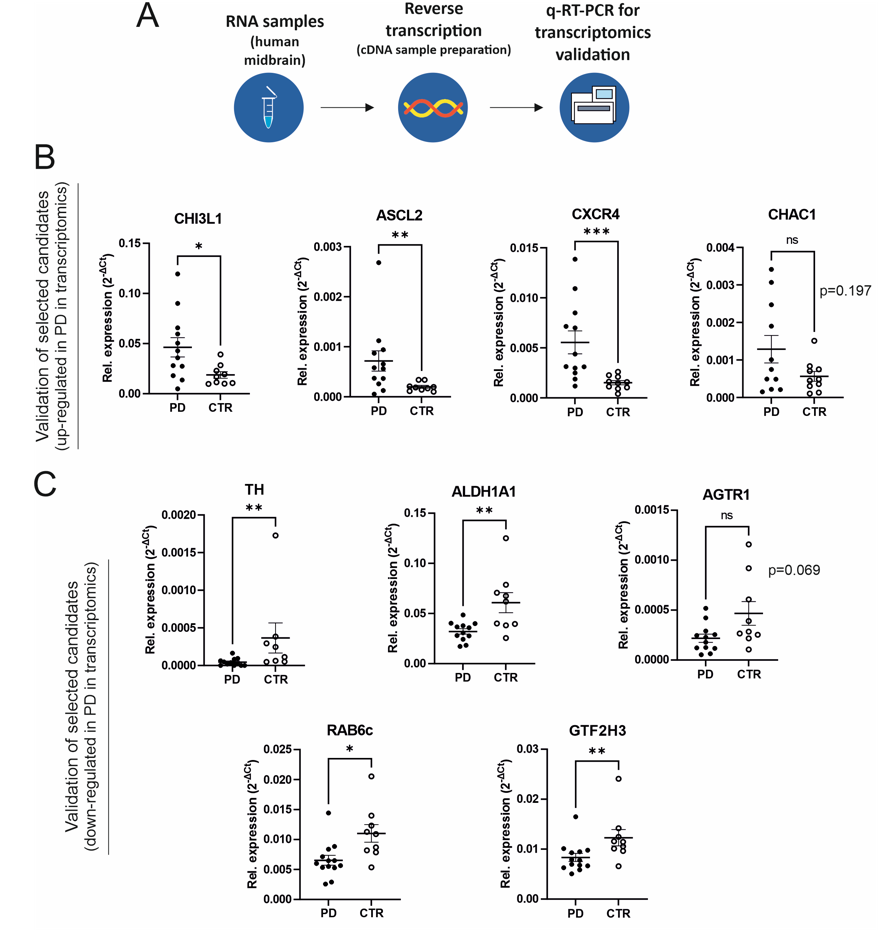
**

**Appendix Figure S8. Experimental setup and validation of transcriptomics results by qRT-PCR. (A)** Experimental setup for the validation of selected genes identified by transcriptomics experiments. Reverse transcription was done with the Quantitect RT Kit (Qiagen) and qRT-PCRs were conducted in the QuantStudio 5 platform. **(B)** Relative expression levels of selected genes found up-regulated in PD patients. **(C)**  Relative expression levels of selected genes found down-regulated in PD patients. Error bars show standard error of the mean. Data analyzed by Mann-Whitney-U test. * p < 0.05, ** p < 0.01, *** p < 0.001.

**
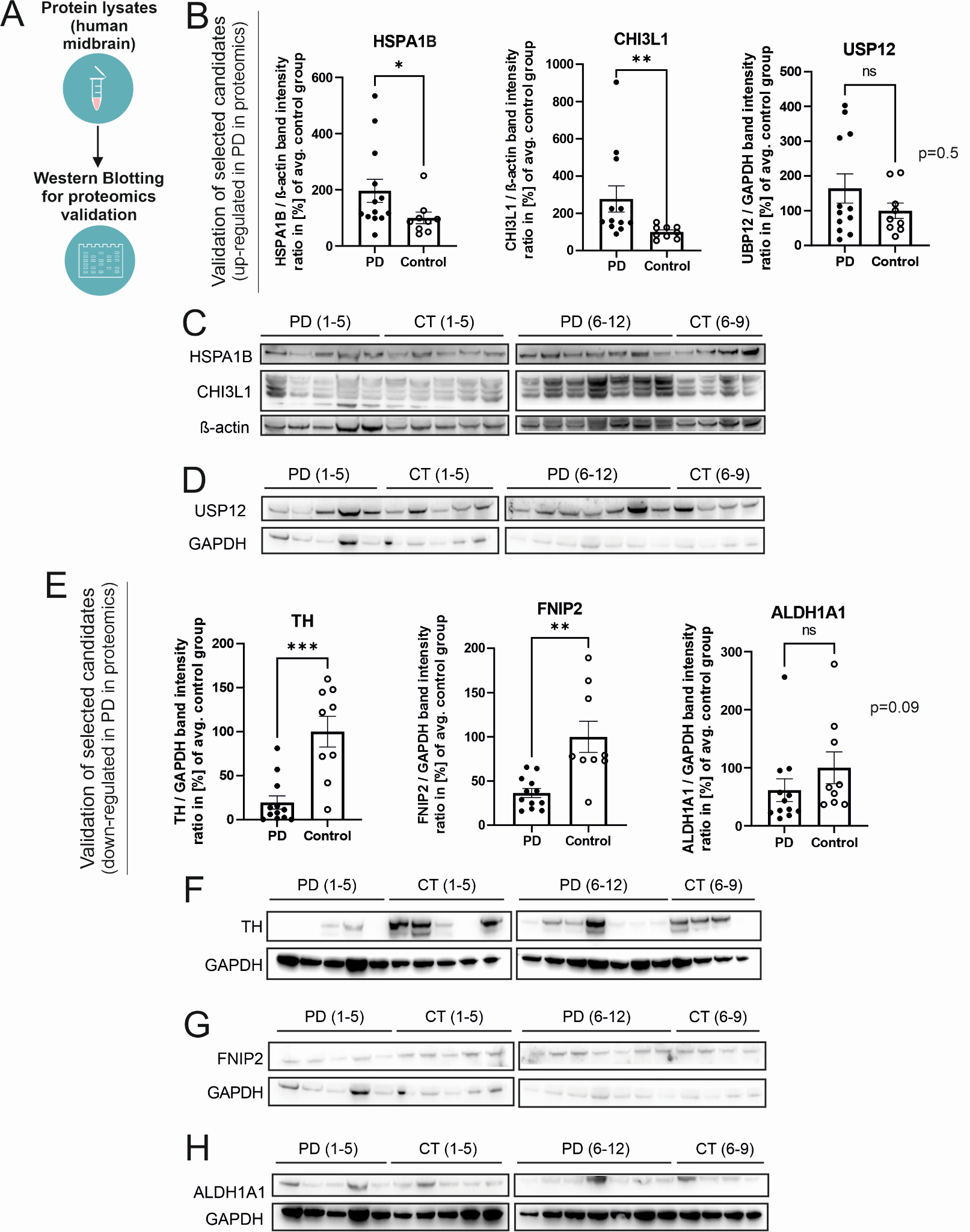
**

**Appendix Figure S9. Experimental setup and validation of proteomics results by Western Blotting. (A)** Experimental setup for the validation of protein candidates. Fresh protein was isolated from postmortem midbrain tissue samples. After protein lysate preparation, Western Blots were conducted with the NuPAGE electroblotting system. **(B)** Relative expression levels of selected proteins found up-regulated in PD patients by proteomics. Bars represent the quantification of band intensities of HSPA1B, CHI3L1 and USP12 (in relation to loading controls). **(C)** and **(D)** depict representative immunoblots of HSPA1B, CHI3L1 and USP12 and the corresponding loading controls, GAPDH and ß-actin. **(E)** Relative expression levels of selected proteins found down-regulated in PD patients by proteomics. Bars represent the quantification of band intensities of TH, FNIP2 and ALDH1A1 (in relation to loading controls). **(F)**, **(G)** and **(H)** depict representative immunoblots of TH, FNIP2 and ALDH1A and the corresponding loading control, GAPDH. Error bars show standard error of the mean. Data analyzed either by Mann-Whitney-U test or unpaired Student’s t-tests. * p < 0.05, ** p < 0.01, *** p < 0.001.

**
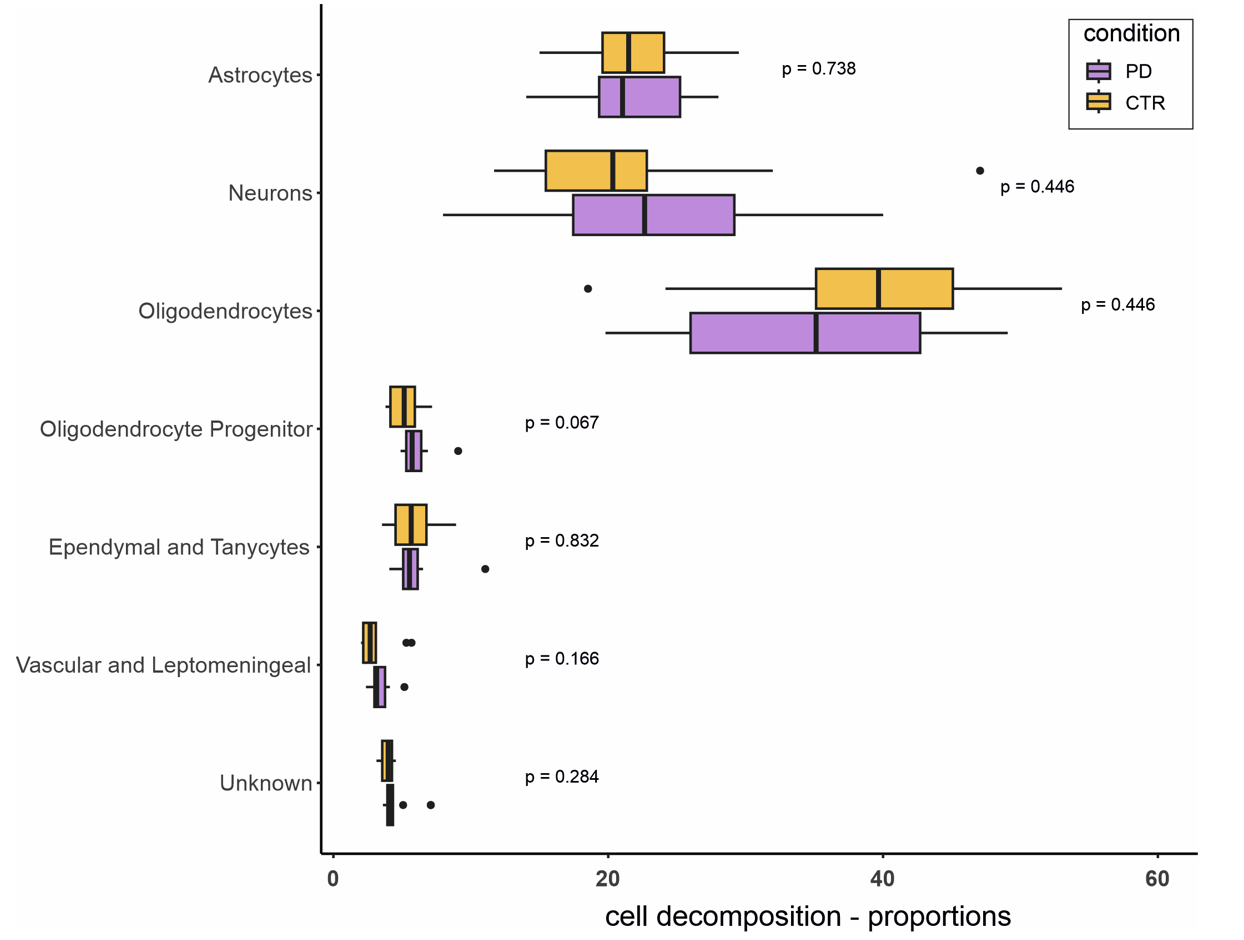
**

**Appendix Figure S10. Cell type deconvolution results of bulk RNA sequencing data with SCADEN framework.** Neuronal cell-type proportions acquired from SCADEN deconvolution (see **Supplementary Methods**). Cellular proportions (in percentage) grouped by cell classes Astrocytes, Neurons, Oligodendrocytes, Oligodendrocyte Progenitor, Ependymal and Tanycytes, Vascular and Leptomeningeal, and Unknown, and disease groups (PD and CTR). Significance within cell-type proportions between biological conditions was tested using Student’s t-test. No significant differences were depicted over the neuronal cell types (smallest p-value > 0.05).

**
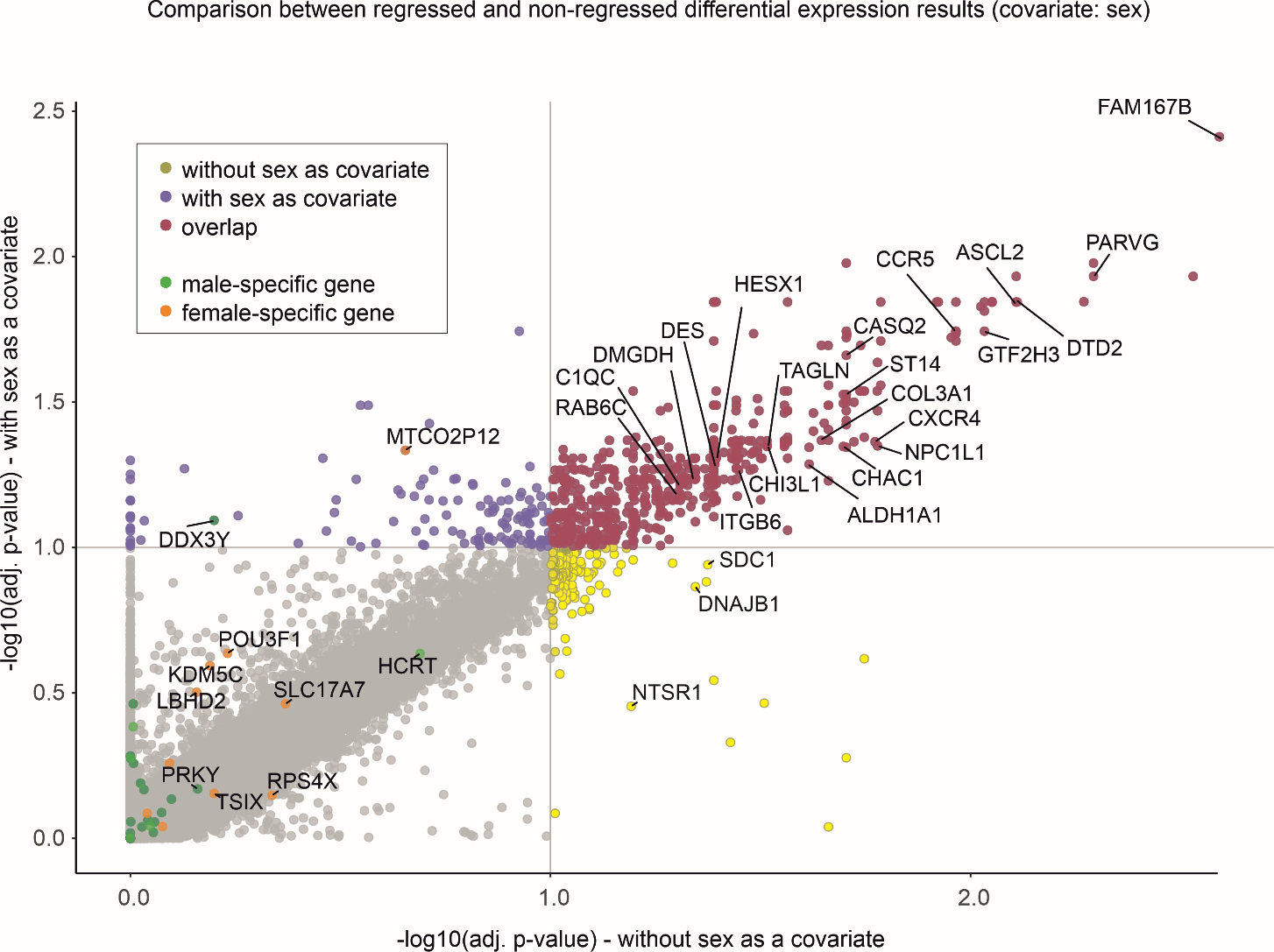
**

Appendix Figure S11**. Comparison of p-adjusted values between controlled and non-controlled differential expression analysis for the sex covariate.** The y-axis represents the -log10(p-adjusted) values for the analyses “with” sex as a covariate, while the x-axis represents -log10(p-adjusted) values for the main analyses (“without” sex as a covariate). The overlap in the differential expression results is highlighted in red, with genes found significantly regulated in both scenarios (p-adjusted < 0.1). For the sex-regresed analyses, only genes which were significant in the differential expression analysis of male vs female subjects are labelled. In yellow: genes uniquely identified by the analyses “without” sex as a covariate; in purple: genes uniquely identified “with” sex as a covariate; in green: male specific genes; in orange; female specific genes. The majority of sex-specific genes are either filtered or not significantly regulated for in both differential expressed scenarios.


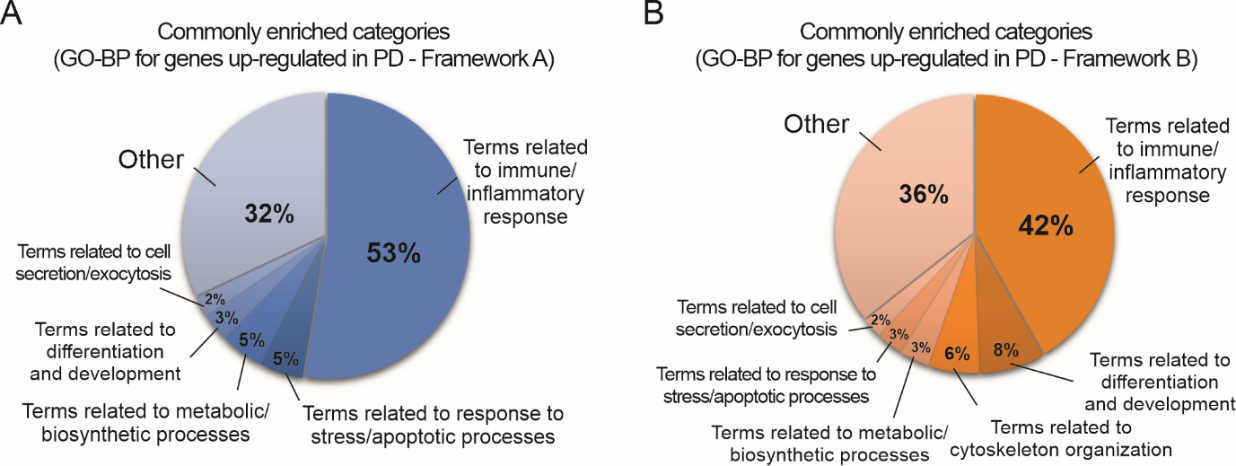


Appendix Figure S12**. Functional enrichment analysis (GO-biological processes and KEGG pathways) of differentially up-regulated genes in PD. (A)** Summary of enriched GO-*biological processes* (GO-BP) categories in the functional annotation for genes up-regulated in PD obtained with framework “A”. **(B)** Summary of enriched GO-*biological processes* (GO-BP) categories in the functional annotation for genes up-regulated in PD obtained with framework “B”.

**
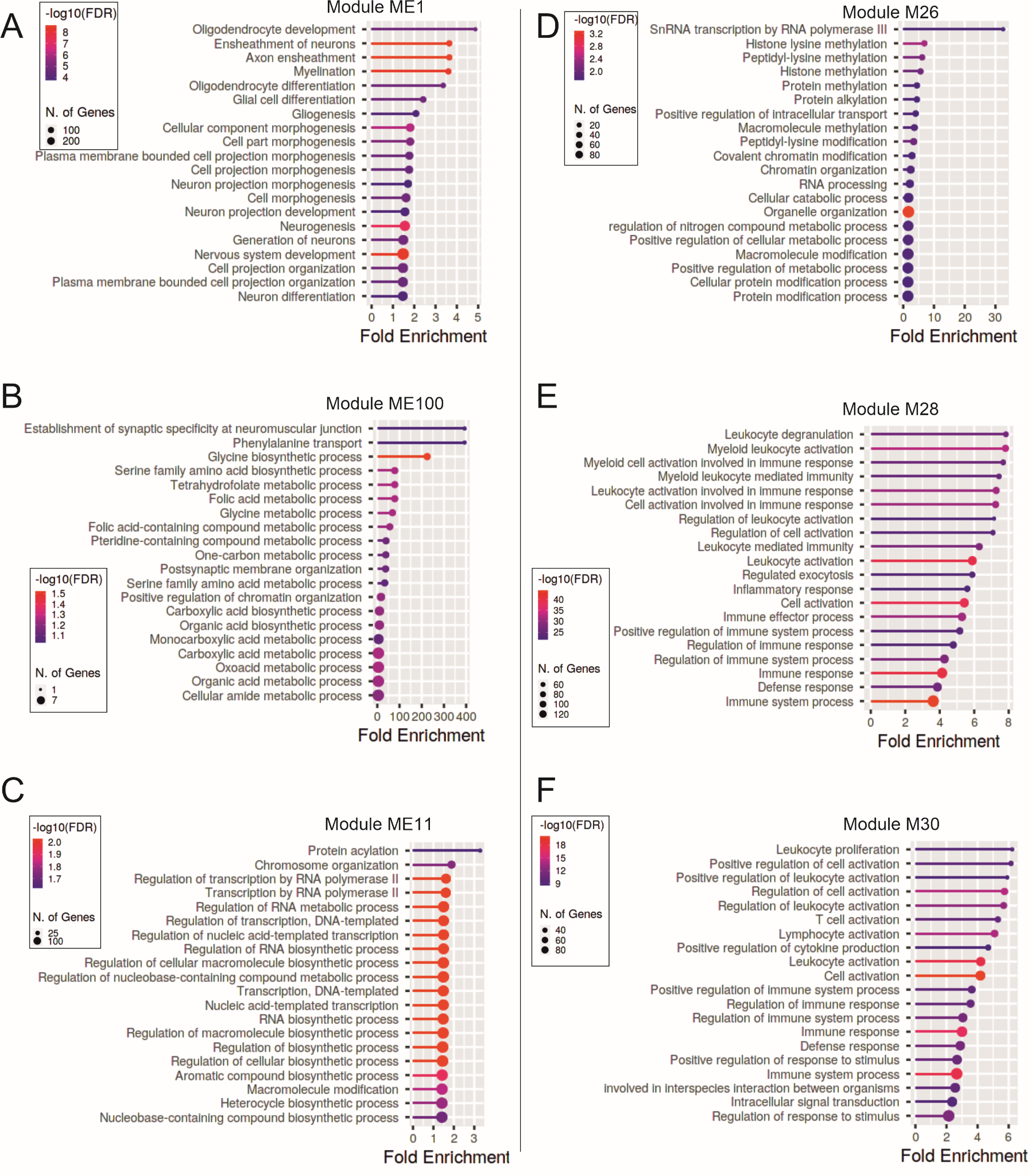
**

**Appendix Figure S13. Gene Ontology - Biological processes (GO-BP) enrichment results for WGCNA identified modules.** Top 20 GO-BP terms enriched for the genes present in each of the significant modules from WGCNA analyses (with p-value < 0.05). Functional annotation was performed using enrichment analysis tools available in ShinyGO [[85],](https://www.zotero.org/google-docs/?5oiOaG) under FDR = 0.1 threshold. Bars represent the fold enrichment for each of the GO-BP terms. Bar colors depict significance of the enrichment (-log_10_FDR values). The size of the ball on the end of the bars denotes the number of genes enriched for each category. Enrichment results depicted for **(A)** Module ME1, **(B)** Module ME100, **(C)** Module ME11, **(D)** Module M26, **(E)** Module M28, **(F)** Module M30.

**
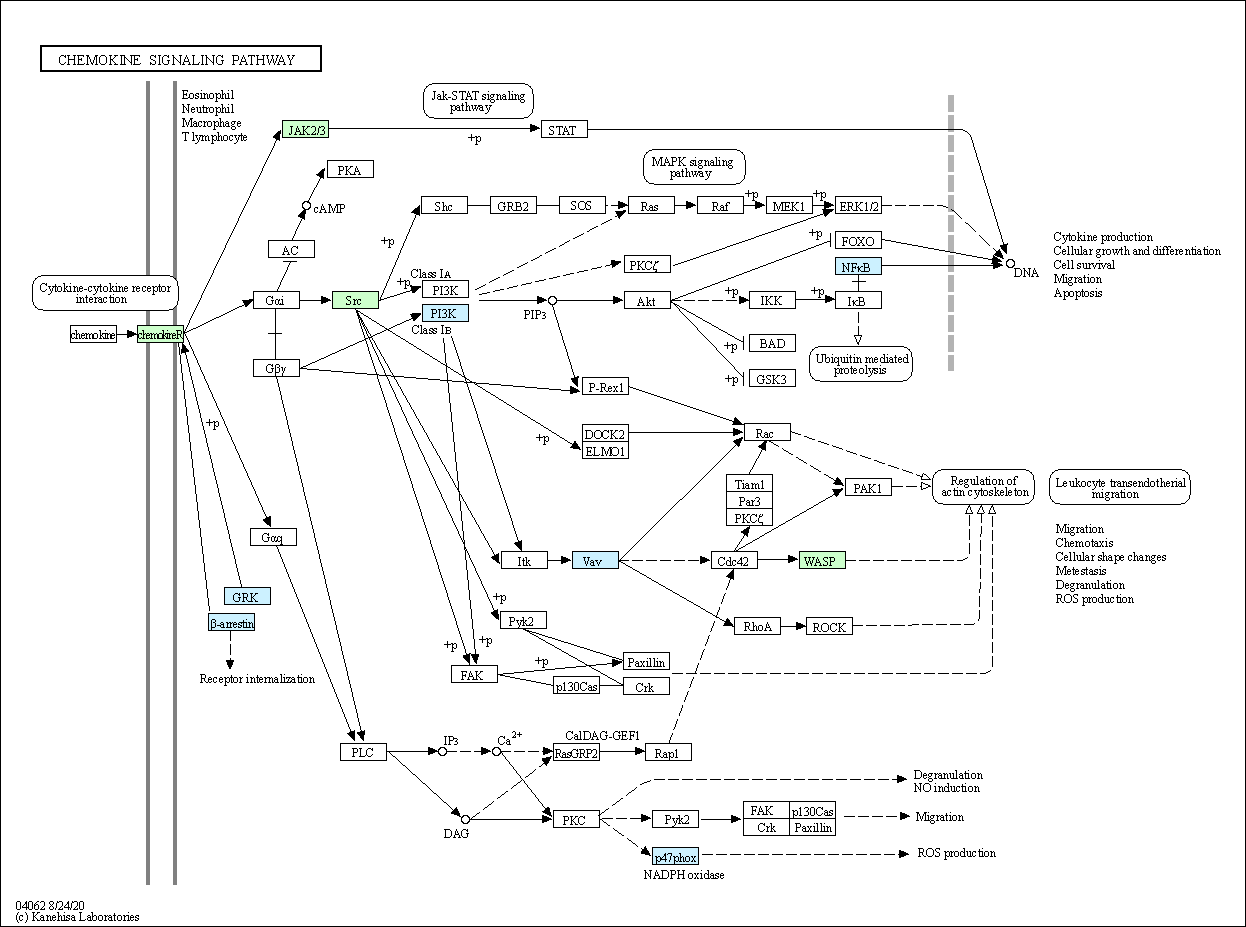
Appendix Figure S14. KEGG human chemokine signaling pathway colored based on the enrichment analysis (FDR < 0.05) of up-regulated significant genes.** Green represents the elements statistically enriched in this pathway for both frameworks “A” and “B” and blue only for framework “A”. Analyses were conducted with ShinyGO v0.61 [[85]](https://www.zotero.org/google-docs/?5oiOaG).


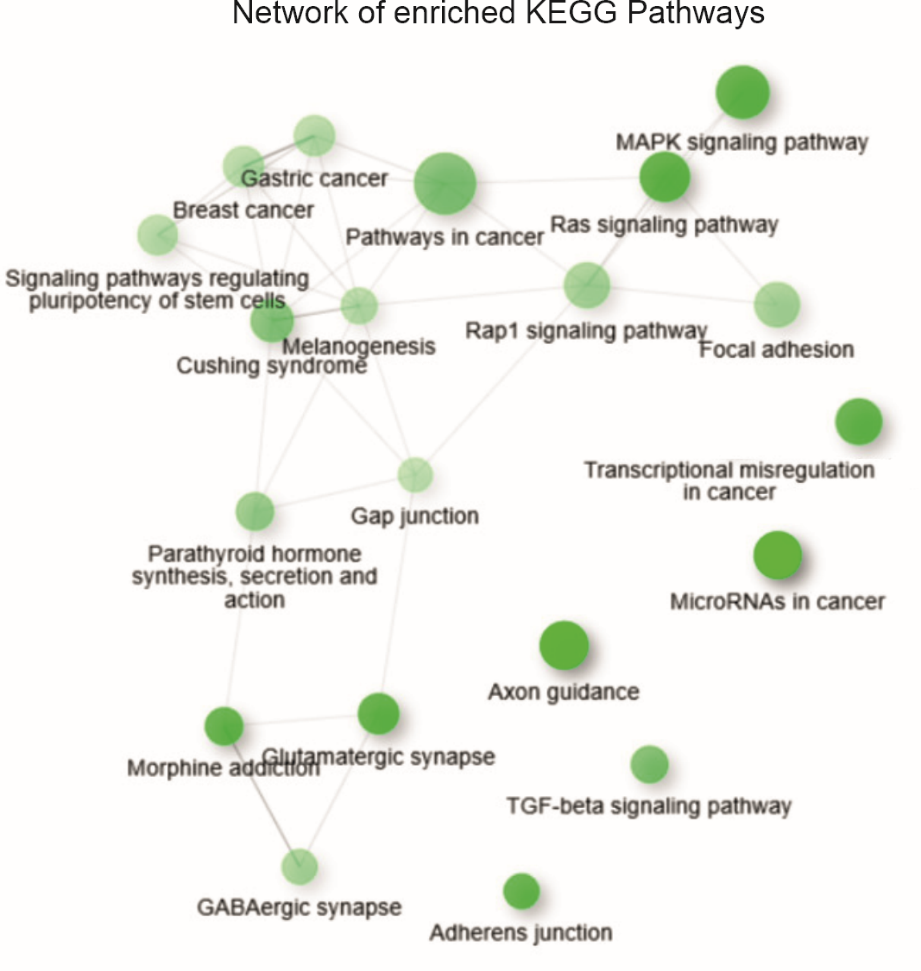


Appendix Figure S15**. Network analysis of *KEGG Pathways* for the genes up-regulated in PD with both analysis frameworks**. Network shows the relationship between enriched terms provided by the ShinyGO v0.61 platform [[85]](https://www.zotero.org/google-docs/?5oiOaG). Connections between pathways denote 20% or more genes shared. Darkness of nodes coded with the level of significance of the enrichment, while node size is representative for the gene set size. Thicker edges represent more overlapped genes.

**
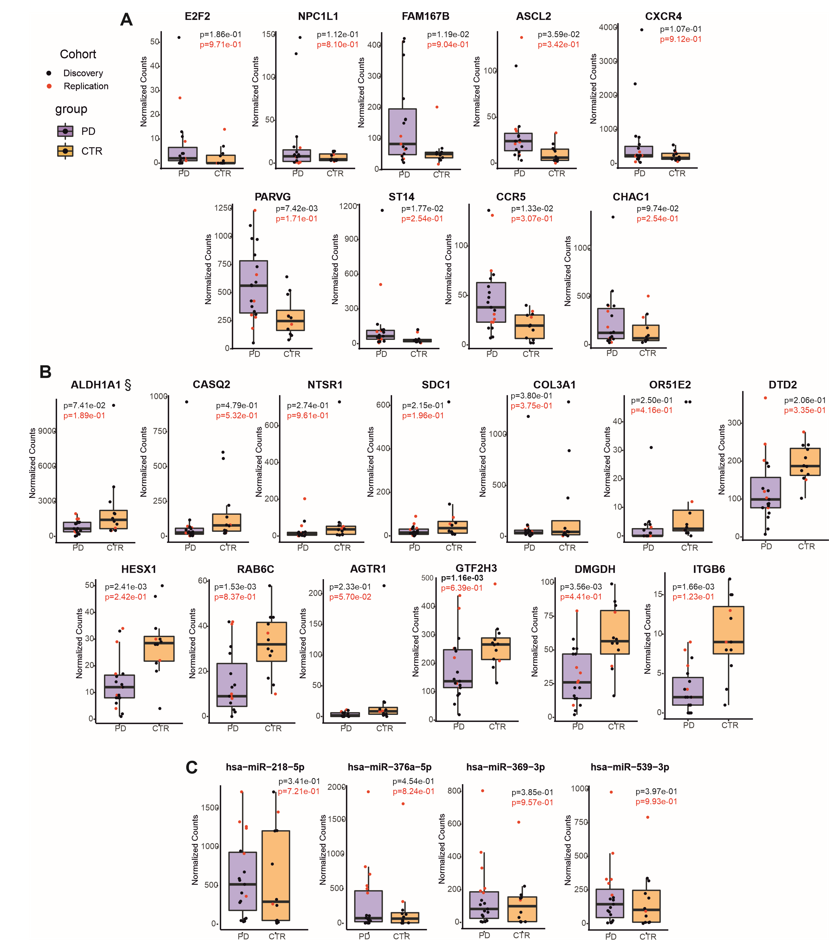
**

**Appendix Figure S16. Barplots of normalized counts for RNA Sequencing experiments, with discovery and validation data cohorts. (A)** Up-regulated genes with a respective miRNA pair (found with both frameworks). **(B)** Down-regulated regulated genes with a respective miRNA pair (found with both frameworks). (§) retinal dehydrogenase 1 (ALDH1A1), to which a valid link was identified after integrating both the miRNA-transcript expression as well as the transcript-protein expression. (C) miRNAs (significant). The p-values were calculated using a two-sided Student’s t-test between PD and CTR samples for each omic analysis.

**
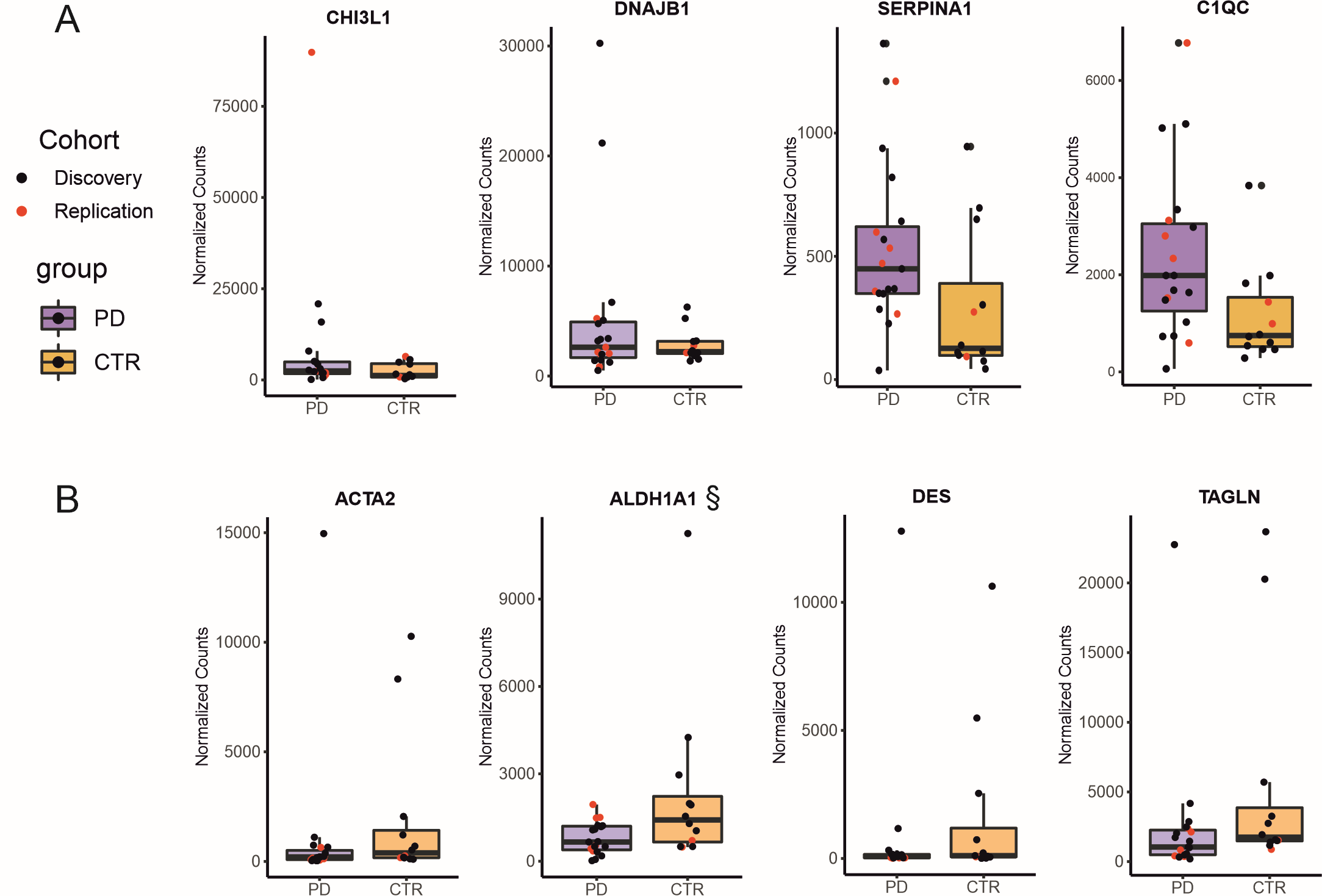
**

**Appendix Figure S17. Transcripts with a valid link in the proteome. (A)** Genes up-regulated in PD with a respective protein pair (found in both frameworks). **(B)** Genes down-regulated in PD with a respective protein pair (found with both frameworks). (§) highlights retinal dehydrogenase 1 (ALDH1A1), to which a valid link was identified after integrating both the miRNA-transcript expression as well as the transcript-protein expression. Data is given in normalized counts, including the replication and discovery cohort and the independent replication cohort. The p-values were calculated using a two-sided Student’s t-test between PD and CTR samples for each omic.
